# Introducing azobenzenes as solid phase peptide synthesis linkers

**DOI:** 10.1039/d6cb00028b

**Published:** 2026-06-08

**Authors:** Connor B. Śmieja, Lizun Xin, Tianhui Tang, Ryan Tan, Martin Lee, Alison N. Hulme

**Affiliations:** a EaStCHEM School of Chemistry, University of Edinburgh David Brewster Rd Edinburgh EH9 3FJ UK Alison.Hulme@ed.ac.uk; b Cancer Research UK Scotland Centre (Edinburgh), Institute of Genetics and Cancer, University of Edinburgh Crewe Road South Edinburgh EH4 2XR UK

## Abstract

Orthogonally cleavable linkers are essential tools in solid-phase peptide synthesis (SPPS). We present a fluorogenic C̲o̲umarin-L̲oaded A̲z̲obenzene (COLAZ) linker for SPPS, which offers base and acid stability and a mild reductive cleavage. We synthesise model peptide WHISKEY with an integrated coumarin label using COLAZ resin and demonstrate that COLAZ and Rink linker technologies may be combined to produce peptides with a latent fluorophore. The biological compatibility of the COLAZ unit is demonstrated *in vitro* by tracking its glutathione-induced cleavage and in live cells by imaging the uptake of an R10 cell-penetrating peptide using two photon fluorescence (TPF) and stimulated Raman scattering (SRS).

## Abstract 2

The C̲o̲umarin-L̲oaded A̲z̲obenzene (COLAZ) linker for SPPS produces peptides with an integrated fluorescent label. Upon its biocompatible cleavage with dithionite or glutathione, the latent fluorophore is revealed enabling live-cell peptide imaging.

## Graphical abstract

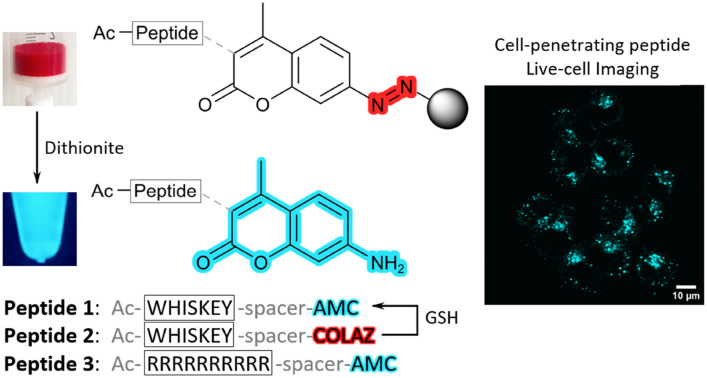

## Introduction

The electrophilic aromatic substitution reaction of aryldiazonium salts to form azobenzenes provides a versatile tool for chemical biology that has found recent use in the affinity-driven labelling of peptide receptors on living cells,^1^ and for profiling post-translational serotonylation.^2^ In a “catch-and-release” mode,^3^ the azobenzene linkage is cleaved under biocompatible conditions using a mild reducing agent such as dithionite. This combined strategy has seen azobenzenes used to chemically modify tyrosine (giving rise to the site-selective introduction of non-canonical amino acids to proteins),^4^ and for a range of affinity chromatography^5,6^ and chemical proteomics applications.^7–10^ We envisaged extending the use of azobenzenes to provide dual-function linkers for solid-phase peptide synthesis (SPPS) applications.

**Fig. 1:**
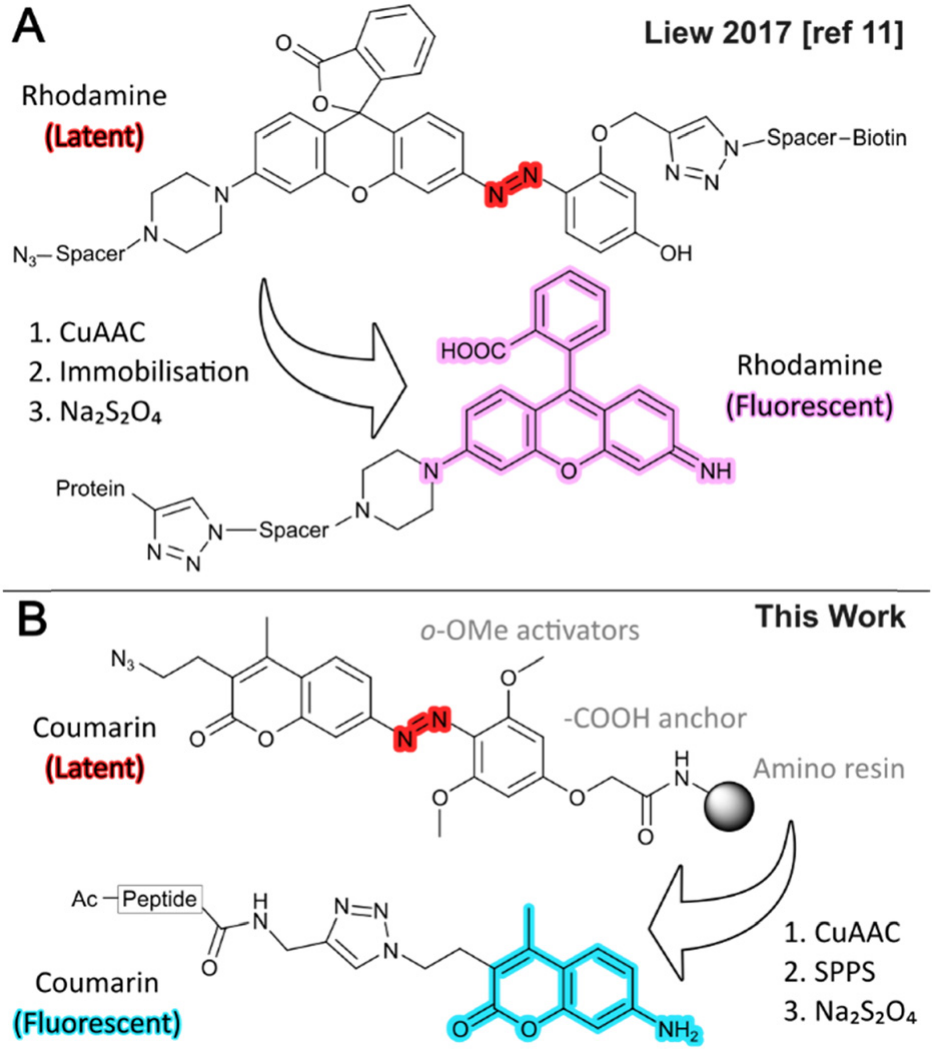
Release of fluorophore-labelled (A) protein and (B) peptide by diazo cleavage.

A study by Liew *et al.* in which the aromatic amine group of a rhodamine dye was incorporated into an azoarene linker for affinity chromatography piqued our interest (Fig. 1A).^11^ Fluorescent dyes are quenched as a result of conjugation to an azo bond, which facilitates nonradiative decay *via* the intersystem crossing (ISC), and *cis*–*trans* interconversion mechanisms;^12,13^ as a result, this azoarene linker is non-fluorescent. However, following CuAAC coupling and affinity pull-down, azo-bond cleavage releases rhodamine-labelled proteins that can be observed directly using in gel fluorescence.^11^ Since the process of orthogonal cleavage from a solid support in proteomics is conceptually similar to the release of peptides from loaded resins in SPPS, we proposed to exploit the photoprotective properties and mild, orthogonal cleavage of the azo bond in this context.

For our proof-of-concept studies into the application of a dye-loaded azobenzene as an SPPS linker, we chose a 7-amino-4-methyl coumarin (AMC) dye (Fig. 1B), to ensure both a straightforward synthesis and compatibility with conventional Fmoc-based SPPS routines. The synthesis and application of this C̲o̲umarin-L̲oaded A̲zobenzene (COLAZ) linker was thus the focus of our approach.

## Results and discussion

### Design of the COLAZ linker

The azobenzene N=N bond resists the strong acidic conditions employed for cleavage of traditional SPPS linkers and the deprotection of the amino acid side-chain protecting groups used in Fmoc-based SPPS.^14,15^ It may instead be cleaved *via* reduction using aqueous solutions of the mild inorganic reductant sodium dithionite. This cleavage reaction can be tuned by the substituents on the flanking arene rings, and is known to be more efficient when methoxy groups are present *ortho*- to the azo moiety;^16,17^ hence, we incorporated a bis-*ortho*-methoxy motif into the COLAZ linker (Fig. 1B). The azo bond is unreactive to the standard copper-catalysed azide–alkyne cycloaddition (CuAAC) “click” conditions that have been used extensively in solid-phase contexts, including protein affinity chromatography.^5,18^ Therefore, we considered an azide to be a versatile choice of handle for the COLAZ linker, as it would permit use in both SPPS and in wider protein applications, while also affording considerable future flexibility to add spacing units of various lengths between the peptide and the dye. Finally, we envisaged the COLAZ linker to have a carboxylic acid anchor, to allow ready attachment to amino-functionalised resins; the same strategy used for the well-established Rink linker.^19^

### Synthesis of the COLAZ linker

**Scheme 1:**
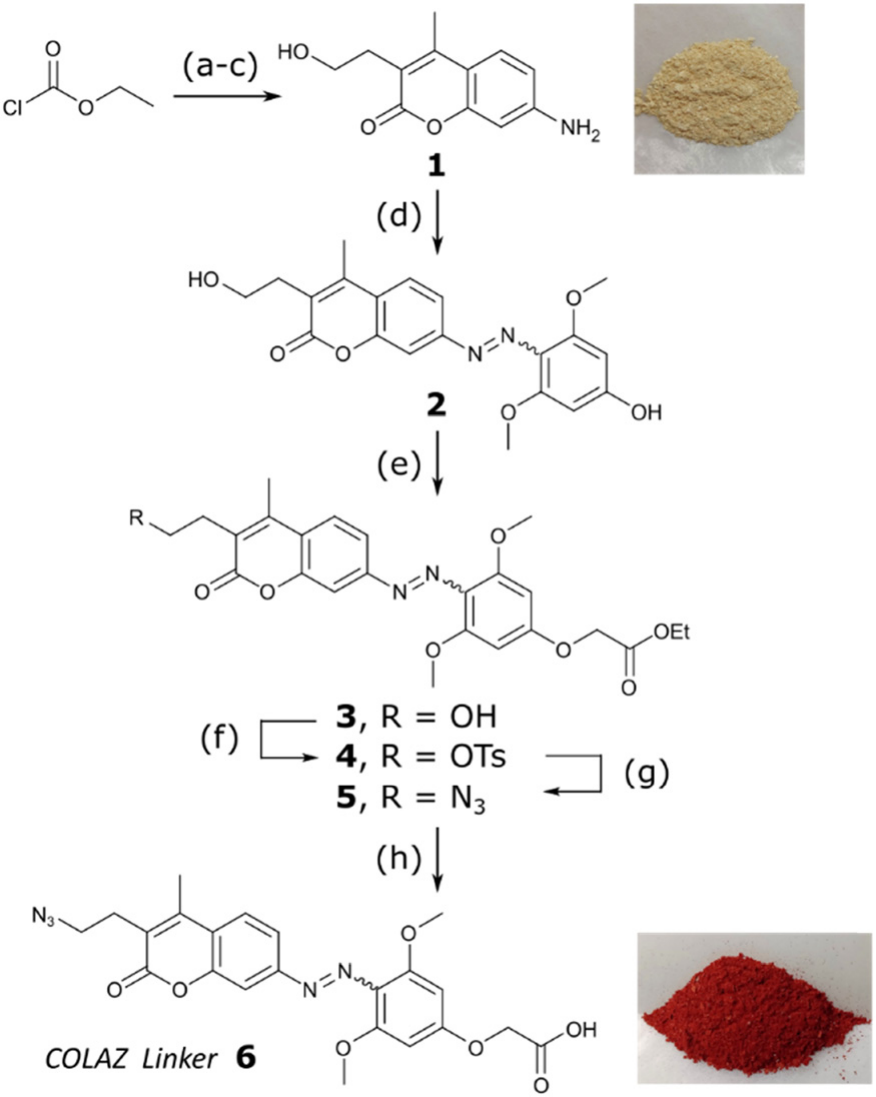
Synthesis of COLAZ linker. Reagents and conditions: (a) *m*-aminophenol, EtOAc, 85 °C, 2 h, quant.; (b) α-acetyl-butyrolactone, 70% H_2_SO_4(aq)_, 0–20 °C, 2 h, 88%; (c) NaOH (12 equiv), H_2_O, 80 °C, 2 h, 99%; (d) i. HCl (0.25 M), NaNO_2_, H_2_O, 0 °C, 30 min; ii. 3,5-dimethoxyphenol, NaOH, K_2_CO_3_, H_2_O, 0–20 °C, 15 min, 88%; (e) ethyl bromoacetate, K_2_CO_3_, DMF, 80 °C, 1 h, 76%; (f) *p*-TsCl, Et_3_N, DMAP (cat.), DCM, 55 °C, 46 h, 56%; (g) NaN_3_, DMF, 65 °C, 6 h, 87%; (h) KOH_(aq)_, THF, 20 °C, 98%.

Synthesis of the COLAZ linker, incorporating all of the desired functionalities, was achieved in 8 steps on a 1.8 mmol scale (Scheme 1). The known 7-AMC derivative 1^20^ was synthesised based on the procedure employed by Maly *et al.*^21^ Azo coupling of 3,5-dimethoxyphenol to 1 was accomplished using conditions reported by our group.^5^ The resultant phenol 2 was then alkylated with ethyl bromoacetate, based on conditions by Li *et al.*,^22^ to give ester 3. The hydroxyethyl group was converted *via* tosylate 4 to azide 5, and finally hydrolysis of the ester gave the COLAZ linker 6. Column chromatography was avoided in all steps except the tosylation, where unreacted starting material could be readily separated and recycled.

As expected, COLAZ linker 6 (Scheme 1) was isolated as a non-fluorescent (Fig. S1), dark red solid that exists as a mixture of *E*/*Z* isomers at room temperature; NMR experiments performed at elevated temperatures enriched the population of 6 in favour of the *E*-isomer (SI file).

### COLAZ linker loading and stability

**Scheme 2:**
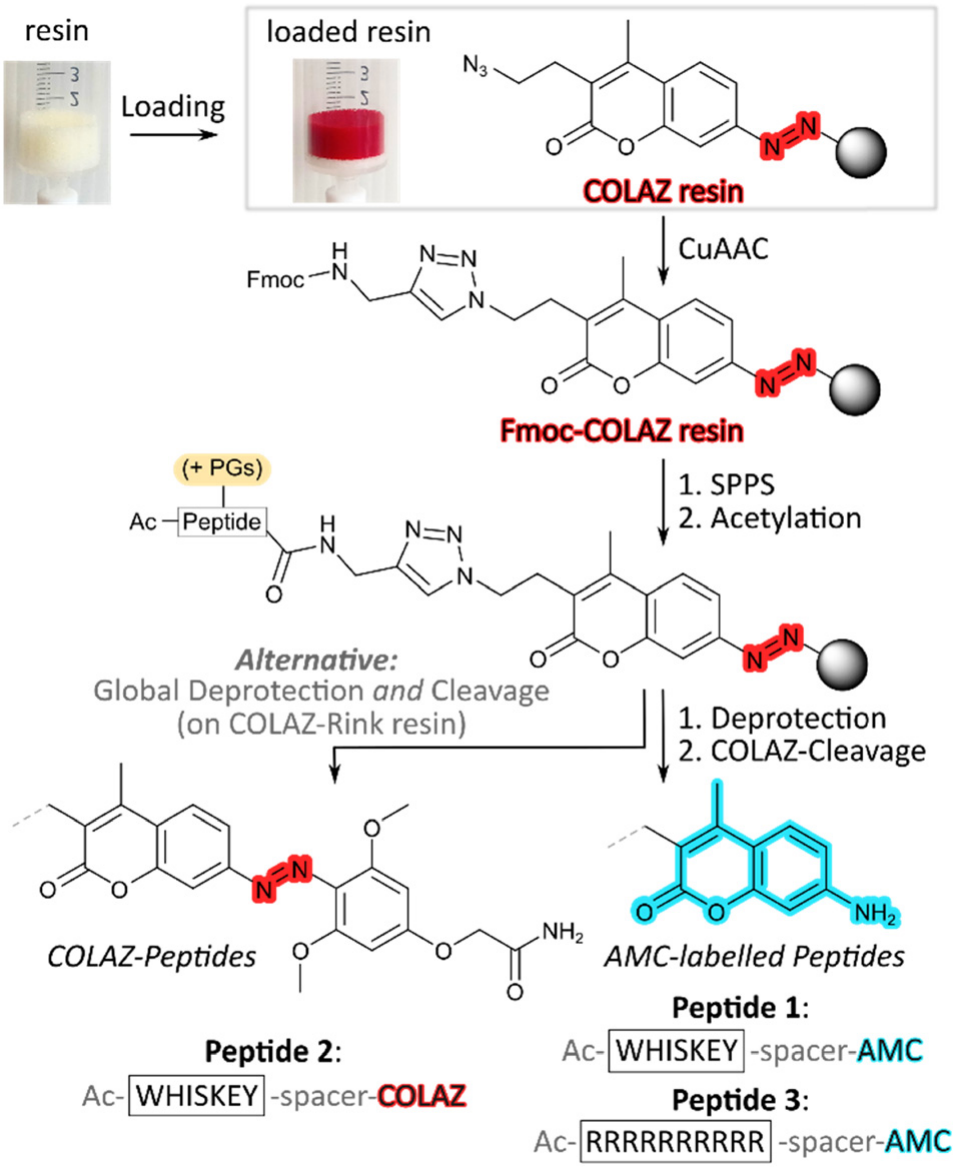
Outline of the SPPS procedure (full details in SI file). COLAZ linker redacted to the coumarin part for simplicity.

TentaGel S resin is generally used for SPPS in combination with an acid-labile linker such as Rink, Wang, or Chlorotrityl. To develop the reductive cleavage protocol for the new linker, we used the simple NH_2_-functionalised TentaGel resin that is supplied without any linker system attached. COLAZ linker 6, which bears an integrated azide handle, was loaded onto TentaGel S NH_2_ resin under microwave irradiation in DMF (70 °C, 30 + 10 min) using DIC/Oxyma coupling reagents to give the linker-functionalised COLAZ resin (Scheme 2 and Fig. S2). To facilitate Fmoc-based SPPS, the azide group was then derivatised *via* CuAAC coupling to *N*-Fmoc-propargylamine to give Fmoc-COLAZ resin (Scheme 2). An iodide-free Cu(MeCN)_4_PF_6_:TBTA catalyst combination prevented the formation of any unwanted 5-iodinated triazole.^23^ The on-resin CuAAC reaction could be followed by tracking the disappearance of the characteristic IR stretch for the azide (∼2100 cm^−1^), which indicated quantitative conversion after 2 h (Fig. S3). This was further confirmed by micro-cleavage of the resin after 2 h and analysis by LC-MS, which showed only the Fmoc-derivatised AMC resulting from CuAAC reaction and reductive cleavage, with no unmodified azido-AMC species detected (Fig. S4).

Next, we demonstrated that COLAZ-cleavage could be applied as a quantitative method for determining the loading of COLAZ linker 6. The COLAZresin was subjected to reductive cleavage (SI file) and the fluorescence of the eluent (containing the cleaved azido-AMC species) was compared against a calibration curve (Fig. S5). A loading of 75% of the resin manufacturer's quote was determined. To compare this with the established method of determining loading using Fmoc-deprotection, Fmoc-COLAZ resin was cleaved using 20% Piperidine in DMF, and the absorbance of the resulting fluorenylmethyl-piperidine adduct in the eluent was measured (SI file). This gave a loading of 70% of the resin manufacturer's quote, which is in excellent agreement with the COLAZ-cleavage loading determination method and also highlights the efficiency of our CuAAC-functionalisation protocol.

We then sought to validate the compatibility of Fmoc-COLAZresin with peptide synthesis, involving subjection to multiple cycles of Fmoc deprotection and amino acid coupling. (Scheme 2). Therefore, we Fmoc-deprotected and acetylated Fmoc-COLAZ resin to give Ac-COLAZ resin, which was exposed repeatedly to Fmoc-deprotection conditions; after 15 consecutive cycles, there was no significant loss of on-resin material (Fig. S6). Side-chain deprotection of all standard acid-labile protecting groups intended for Fmoc-based SPPS (Table 1) can be achieved by soaking the peptide resin in 95% TFA (with 2.5% H_2_O and 2.5% TIPS as scavengers). Using standard acid-labile linkers such as Rink, Wang or Chlorotrityl, the deprotected peptide itself would also be cleaved from the resin under these conditions. We tested the stability of Ac-COLAZ resin over seven one-hour cycles of exposure to these strongly acidic conditions (Fig. S7) and found that the resin was stable to these harsh conditions, in contrast to standard acid-labile SPPS linkers. This means that in COLAZ-based SPPS, side-chain deprotected peptides are retained on-resin, which presents two main advantages. Firstly, further side-chain derivatisation could be performed prior to COLAZ-cleavage. Secondly, there is no need to retrieve a deprotected peptide product from concentrated TFA. Instead, COLAZ-cleavage is performed under mild conditions by soaking the resin in 0.25 M sodium dithionite solution in 1 : 1 *t*BuOH:water; the resulting eluent is then simply lyophilised to obtain a crude peptide product. In addition, COLAZ-cleavage yields peptides that are labelled quantitatively with a fluorescent 7-amino-4-methyl-coumarin (AMC) derivative through a spacer unit, ultimately linked *via* an amide bond to the peptide C-terminus (Scheme 2).

**Table 1:** Acid-labile protecting groups (PGs) compatible with the COLAZ linker

PG functionality	Amino acids^a^
*tert*-Butyloxycarbonyl (Boc)	W,K,H
*tert*-Butyl ether (*t*Bu)	S,T,Y
*tert*-Butyl ester (*t*Bu)	D,E
2,2,4,6,7-pentamethyldihydrobenzofuran-5-sulfonyl (Pbf)	R
2,2,5,7,8-pentamethylchroman-6-sulfonyl (Pmc)	R
Triphenylmethyl (Trt)	H,N,Q,C,W

a Amino acids given in single letter code.

### Peptide synthesis

WHISKEY has been proposed as a model peptide for SPPS reaction development, as it represents the majority of the chemical functionality found in natural amino acids.^24^ The Fmoc-mediated SPPS of WHISKEY also employs amino acid building blocks bearing a range of side-chain protecting groups, namely *N*-Boc (W,K), *N*-Trt (H), *t*Bu ether (S,Y), and *t*Bu ester (E). Synthesis of WHISKEY (Peptide 1, Scheme 2) using COLAZ resin was successfully performed on mg scale on a commercial peptide synthesiser, further demonstrating the stability of the COLAZ linker to both the Fmoc-deprotection and side-chain deprotection conditions. Crude COLAZ-cleaved Peptide 1 was readily purified by preparative RP-HPLC to ≥90% purity in 32% overall yield, and its identity was confirmed by MALDI-MS (SI file).

**Fig. 2:**
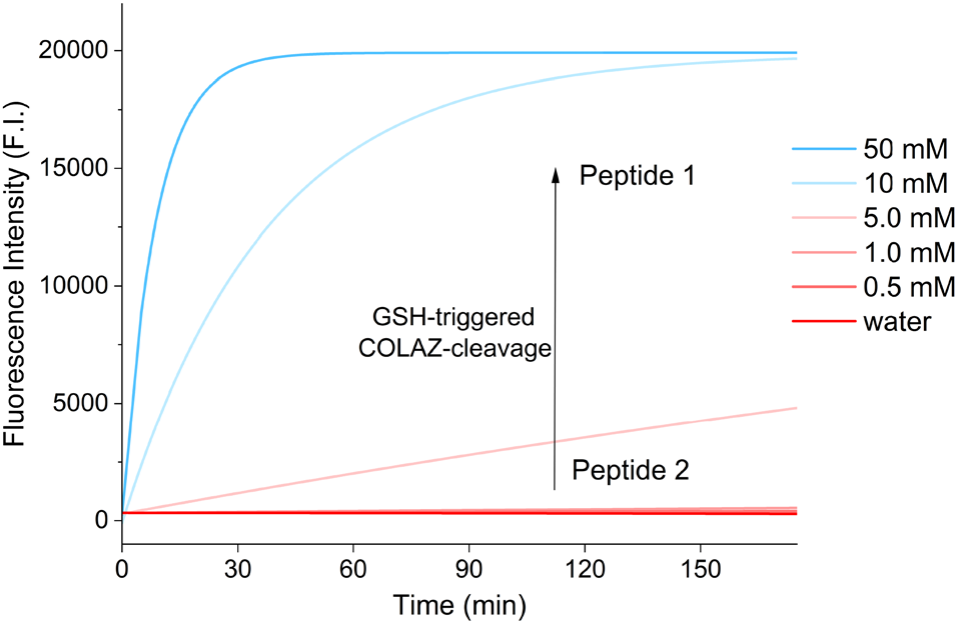
GSH concentration-dependent cleavage of the COLAZ unit in Peptide 2 in PBS solution. The reaction was monitored in real-time by measuring the fluorescence intensity (*λ*_ex_ = 365 nm, *λ*_em_ = 450 nm, 5 min intervals) of the AMC unit in the cleavage product (Peptide 1).

Next, we investigated whether the COLAZ linker could be combined with the acid-labile Rink-linker to obtain side-chain deprotected peptides in solution bearing an intact COLAZ unit. Such peptides could then be subjected to orthogonal COLAZ-cleavage at a later stage to activate the integrated fluorescent label. Microwave-promoted loading of COLAZ linker onto commercial Rink Amide AM resin, followed by on-resin CuAAC click reaction as described previously, gave Fmoc-COLAZ-Rink AM resin, loaded to 68% of the manufacturer's stated loading, consistent with our previous results (SI file). The WHISKEY peptide was synthesised on this COLAZ-functionalised Rink resin and subsequent application of side-chain deprotection conditions led to concomitant cleavage of the Rink linker and peptide release. Preparative RP-HPLC afforded a WHISKEY peptide bearing a COLAZ unit (Peptide 2, Scheme 2), in ∼88% purity and 12% overall yield (SI file). We attribute the lower yield achieved using this resin to difficulties in precipitating the crude peptide from ether, which highlights the practical advantages of employing the COLAZ linker as a means to separate the deprotection and resin cleavage steps. The COLAZ unit in Peptide 2 acts as a latent AMC fluorophore, which can be revealed upon reductive cleavage. To highlight the biological applicability of peptides synthesised using this approach, we explored whether COLAZ-cleavage could occur with reduced glutathione (GSH) as well as sodium dithionite (Fig. 2) (SI file). The COLAZ moiety in Peptide 2 cleaved rapidly (20–60 min) in the presence of 10–50 mM GSH, a concentration comparable to biologically relevant intracellular ranges.^25^ In contrast, Peptide 2 was stable at the lower concentrations of GSH typical of serum (<0.5 mM).

Finally, we synthesised the polyarginine peptide R10 with a C-terminal AMC label using the COLAZ linker (Peptide 3, Scheme 2). Polyarginine-containing peptides have been widely studied as potential drug delivery vehicles, and the relationship between their structure and uptake efficiency across different cell types has been investigated.^26,27^ However, these peptides have generally been labelled at the peptide *N*-terminus, or on their sidechains.^28,29^Peptide 3 was synthesised on Fmoc-COLAZresin on mg scale using the same protocols as for Peptide 1, and was purified by preparative RP-HPLC to 90% purity in 17% yield (SI file). The identity of Peptide 3 was confirmed by MALDI-MS, with the only impurities identified as traces of the R9 and R8 peptides arising from incomplete coupling, the presence of which would not be detrimental to subsequent imaging studies.

### Cellular imaging

**Fig. 3:**
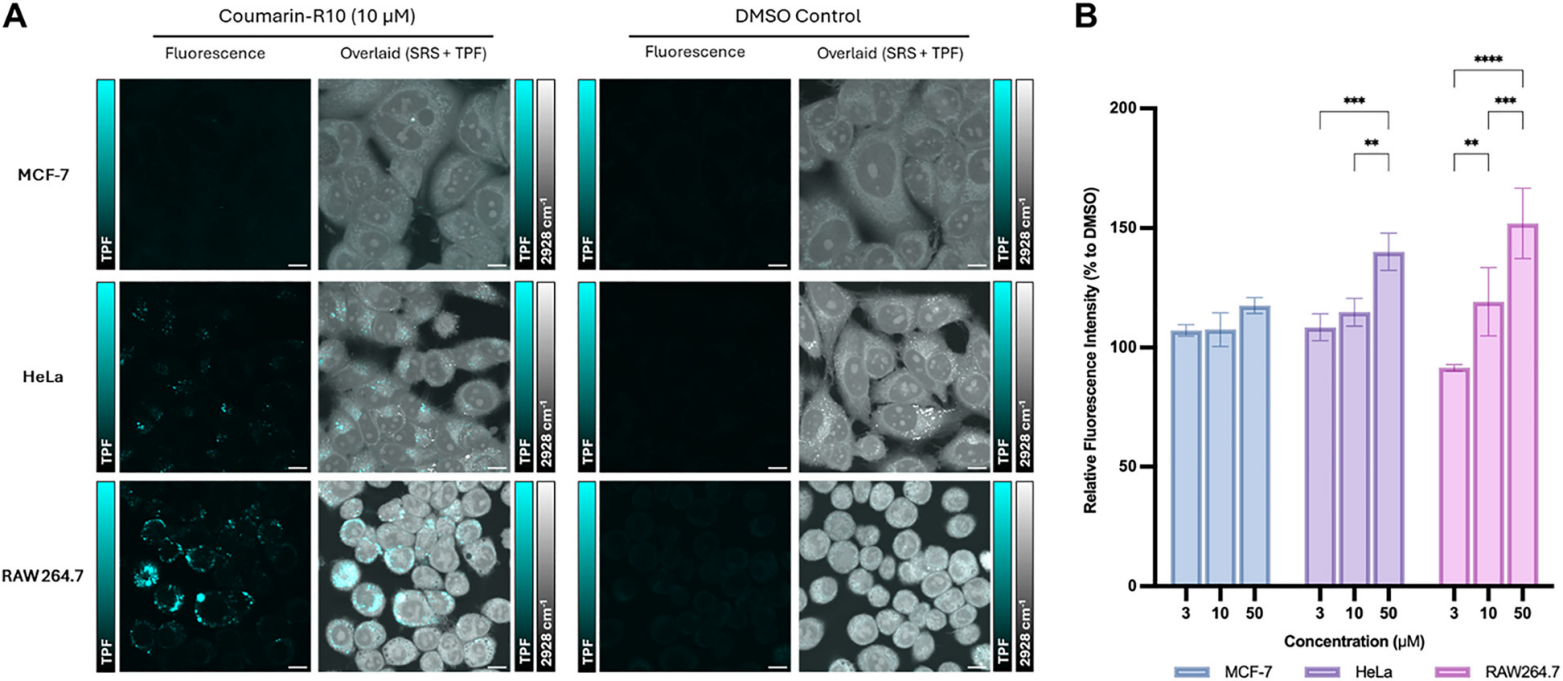
TPF and SRS microscopy of coumarin-labelled R10 Peptide 3 in MCF-7, HeLa and RAW264.7 cells following incubation (10 µM, 1 h) alongside DMSO controls. A (left of pair): TPF images (*λ*_ex_ = 740 nm; emission collected 520–552 nm); (right of pair): SRS images (2928 cm^−1^) overlaid with TPF images. Scale bars = 10 µm. B Relative cellular uptake quantified from fluorescence microscopy data shown in Fig. S6. (Normalised to DMSO, two-way ANOVA pairwise comparison, statistical significance is denoted as follows: ** *p* < 0.01, *** *p* < 0.001, **** *p* < 0.0001.)

The AMC fluorophore is typically excited in the UV range at ∼344 nm, giving rise to blue fluorescence between 440–470 nm. Although AMC is frequently used in enzyme assay readouts, this excitation wavelength is not ideal for live cell imaging as UV irradiation can damage cells and induces background autofluorescence from endogenous fluorophores such as NADH and collagen.^30^ However, the two-photon fluorescence (TPF) of AMC can be induced by excitation at 740 nm, which is compatible with cellular imaging, and can also be achieved using the IR laser of a coherent Raman microscope.^31^ We thus evaluated the uptake of AMC-labelled R10 Peptide 3 in MCF-7, HeLa and RAW 264.7 macrophage cell lines, using a combination of TPF and coherent Raman imaging. After 1 h incubation, TPF imaging of the AMC fluorophore in Peptide 3 was conducted with *λ*_ex_ = 740 nm, and emission collected across 520–552 nm using a 510/84 bandpass filter. Concomitant label-free imaging of the C–H vibrations of proteins at 2928 cm^−1^ using stimulated Raman scattering (SRS) microscopy, was used to provide cellular registration (Fig. 3A).

Peptide 3 showed a dose-dependent uptake in two of the cell lines, with fluorescence intensities across a field of view increasing from 3 to 50 µM (Fig. 3B). RAW 264.7 macrophages exhibited the highest uptake of all three cell types, with a 150–160% increase at 50 µM relative to the DMSO control; this is consistent with their higher endocytotic activity.^28^ The SRS- fluorescence overlays show that Peptide 3 is concentrated within the cytoplasm, but excluded from the cell nuclei. In both HeLa cells and RAW 264.7 macrophages, the fluorescence is predominantly localised at the Golgi apparatus, which is also consistent with an endosome-like uptake mechanism. In contrast, MCF-7 cells appear not to be as efficient in their uptake of Peptide 3.^26^ Together, these data suggest that the COLAZ C-terminal labelling strategy preserves the canonical uptake behaviour and subcellular distribution of cationic cell penetrating peptides such as R10 in different cell lines.^25,27^

## Conclusions

The COLAZ linker is the first example of an azobenzene designed for use as an orthogonally-cleavable linker in Fmoc-based SPPS. It can be readily synthesised on a >1 mmol scale in only 8 steps, with a 28% overall yield and just a single chromatographic purification. The linker design incorporates an azido group to allow the spacing unit between the peptide C-terminus and the AMC fluorophore to be readily varied, through CuAAC reactions with different alkyne substrates. Furthermore, we envisage that the integration of other fluorescent dyes bearing an aromatic amine functionality could expand the future range of peptide C-terminus labels.

The compatibility of the COLAZ linker with standard Fmoc-SPPS conditions has been demonstrated through the synthesis of the WHISKEY peptide sequence in Peptide 1. The COLAZ linker is shown to have several practical advantages: loading and cleavage steps can be followed visually in a qualitative manner by observing the obvious colour changes; and cleavage solutions of crude peptides only require direct lyophilisation rather than concentration, precipitation and trituration of solutions of highly concentrated TFA. This makes the COLAZ linker a practical alternative to standard acid labile resins, particularly when a fluorescent label is desired. Future studies will explore the use of the COLAZ linker approach to cleave peptides that are still side-chain protected, to allow their use as fragments in the assembly of larger peptides before global side-chain deprotection.

Combining the COLAZ linker and Rink linker, the entire COLAZ functionality can be transferred to cleaved, deprotected peptides, as demonstrated for Peptide 2. The reductive cleavage of the COLAZ moiety in Peptide 2 to reveal the latent AMC fluorophore can be achieved using ≥5 mM GSH. This suggests that COLAZ-modified peptides such as Peptide 2 could be useful as GSH-activated intracellular probes for chemical biology applications. The fluorescent R10-containing Peptide 3 was also successfully obtained on mg scale using the COLAZ linker, and its AMC fluorophore was used to assess the endocytosis of this cell-penetrating peptide using TPF imaging in combination with SRS microscopy.

Overall, this study demonstrates the utility of our COLAZ linker as a convenient tool to produce peptides labelled at their C-terminus with either a coumarin or latent AMC functionality, with clear potential applications in peptide synthesis, chemical biology and cellular imaging.

## Author contributions

CBS. conceptualisation, investigation, methodology, validation, writing – original draft, review & editing. LX conceptualisation, investigation, methodology, validation, writing – original draft, review & editing. TT investigation. RT investigation. ML methodology, validation, writing – review & editing. ANH conceptualisation, funding acquisition, project administration, writing – review & editing.

## Conflicts of interest

There are no conflicts to declare.

## Supplementary Material

CB-OLF-D6CB00028B-s001

## Data Availability

The data supporting this article have been included as part of the supplementary information (SI). Supplementary information: experimental details & NMR spectra for COLAZ linker synthesis; experimental details, HPLC & MS spectra for peptide synthesis; Fig. S1–S6; details of cell culture & imaging. See DOI: https://doi.org/10.1039/d6cb00028b.
