# Supplementary material for: Introducing azobenzenes as solid phase peptide synthesis linkers
Source: RSC Chem Biol. 2026 Jun 8. Online ahead of print. doi: 10.1039/d6cb00028b (PMC13255771; doi:10.1039/d6cb00028b)
Supplement: CB-OLF-D6CB00028B-s001 [file CB-OLF-D6CB00028B-s001.pdf]

Supplementary Information (SI) for RSC Chemical Biology.
This journal is © The Royal Society of Chemistry 2026

Supplementary Information: Introducing Azobenzenes as Solid Phase Peptide Synthesis Linkers

Connor B. Śmieja,[a,†] Lizun Xin,[a,†] Tianhui Tang,[a] Ryan Tan,[a] Martin Lee[b] and Alison N. Hulme[a,*]

[a.] EaStCHEM School of Chemistry, University of Edinburgh, David Brewster Rd, Edinburgh EH9 3FJ, U.K.
[b.] Cancer Research UK Scotland Centre (Edinburgh), Institute of Genetics and Cancer, University of Edinburgh, Crewe Road South, Edinburgh, EH4 2XR, U.K.

Contents

COLAZ Linker Synthesis: 2
- General Considerations: 2
- COLAZ Linker Compound Synthesis and Characterisation: 3
- NMR Spectra: 9

COLAZ Peptide Synthesis: 17
- General Considerations: 17
- Synthesis: 18
- UPLC Chromatograms, Peptides 27
- MALDI Spectra, Peptides 28

Biorthogonal Cleavage of Peptide 2 30

Cell Culture and Imaging 31

† These authors contributed equally to this work.

COLAZ Linker Synthesis:

General Considerations:

Starting materials and reagents were obtained from commercial suppliers and used without further purification. Saturated aqueous (sat. aq.) solutions of inorganic salts were freshly made for work-up procedures. Thin layer chromatography (TLC) was performed using Merck silica gel aluminium-backed plates (Kieselgel 60 F254, 0.2 mm thickness) with visualisation using ultraviolet light (254 nm or 365 nm), including chemical staining where specified. TLC data is quoted as a retention factor (R_f) with the solvent system quoted as a % or v:v ratio.

Infra-red spectra (IR) were recorded neat on a Thermo Scientific Nicolet Summit Spectrometer. Characteristic absorbances (≥ 1500 cm^{-1}) are reported in wavenumbers (cm^{-1}). Fluorescence and absorbance data were acquired using a BMG Labtech CLARIOstar Plus plate reader.

NMR Spectra were recorded using Bruker AV400, AV500, PRO500 or AV600 at the stated frequency, at ambient temperature (≈300 K) unless otherwise stated. The ^{1}H NMR data are recorded as follows: Chemical shift is quoted in ppm on the δ scale relative to residual solvent peaks unless otherwise stated. Standard abbreviations are used to describe the peak multiplicity (e.g., s = singlet, d = doublet, t = triplet, q = quartet, m = multiplet, br = broad). Coupling constants (*J*) are quoted to the nearest 0.1 Hz. NMR signals were assigned by displaying the observed atom in italic, for example aryl quaternary carbon (Ar*C*), aryl proton (ArC*H*), and equivalent methylene protons (CH_2). Appropriate coefficients (i.e., 2CH_3) are given for symmetrical compounds.

High resolution mass spectrometry (HRMS) analysis was performed via the School of Chemistry's MS facility using matrix assisted laser desorption ionisation (MALDI). Samples were run using a 12T SolariX FT-ICR MS equipped with MALDI ionization using a SmartBeam II UV laser (Bruker Daltonics, Bremen, Germany) in positive ion mode. Samples were co-crystalised on a Bruker MTP 384 ground steel plate with CHCA (10 mg/mL). A m/z range of 150 – 3000 was acquired at 30 % laser power.

LC-MS samples were analysed on an Agilent 6230 LC / TOF walk-up system at the school of chemistry's MS facility. The HPLC method used a Zorbax extend-C18 Column (1.8 Å, 2.1 x 50 mm). The total run time was 9.0 min at a flow rate of 500 µL min^{-1} and the column was maintained at 45 ± 1 °C. A binary solvent system was used; A = H_2O + 0.1% FA and B = MeCN + 0.1% FA. The elution program was isocratic for 0.5 min (95A : 5B) then a linear gradient from 0.5 min to 5.0 min (5A : 95B). The ratio was kept at isocratic until 6 mins (5A : 95B) before recovery of the initial conditions over 0.1 min and equilibration over 2.9 mins.

COLAZ Linker Compound Synthesis and Characterisation:

3-*N*-(Carboxyethyl)aminophenol (3-NCAP, **S1**)

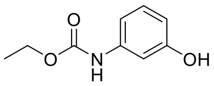

Ethyl acetate (30 mL) was added to 3-aminophenol (5.00 g, 46.1 mmol, 2 equiv.) in a 2-neck 100 mL rbf fitted with a condenser and a septum side-neck and heated with stirring at reflux (85 °C) until the solid was fully dissolved (30 min). Ethyl chloroformate (2.20 mL, 23.0 mmol, 1 equiv.) was added dropwise over 15 min, then the reaction was heated further at reflux (2 h). The reaction was cooled in an ice bath and the white precipitate was removed by filtration under vacuum, washing with ice-cold ethyl acetate (2 x 15 mL). The filtrate was concentrated *in vacuo* to afford the product **S1** as a white solid (4.17 g, 23.0 mmol, quant.).

TLC (SiO_2, 5% MeOH in DCM) R_f = 0.60. **IR** (cm^{-1}) 1684 (C=O). **^{1}H NMR** (500 MHz, Methanol-*d*$_4$) δ 7.05 (dd, *J* = 8.1, 8.1 Hz, 1H, Ar*H*), 7.01 (dd, *J* = 2.2, 2.2 Hz, 1H, Ar*H*), 6.81 (ddd, *J* = 8.1, 2.2, 0.9 Hz, 1H, Ar*H*), 6.45 (ddd, *J* = 8.1, 2.2, 0.9 Hz, 1H, Ar*H*), 4.16 (q, *J* = 7.1 Hz, 2H, C*H*$_2$), 1.29 (t, *J* = 7.1 Hz, 3H, C*H*$_3$). **^{13}C NMR** (126 MHz, Methanol-*d*$_4$) δ 158.96 (Ar*C*), 156.04 (*C*=O), 141.37 (Ar*C*), 130.46 (Ar*C*H), 111.11 (Ar*C*H), 110.92 (Ar*C*H), 107.01 (Ar*C*H), 61.78 (*C*H$_2$), 14.92 (*C*H$_3$). **HRMS (MALDI)** (M = $C_9H_{11}NO_3$) calc. $[M+H]^+$ = 182.0812, found = 182.0810.

7-*N*-(Carboxyethyl)amino-3-hydroxyethyl-4-methylcoumarin (**S2**)

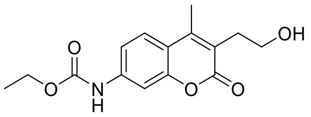

3-*N*-(Carboxyethyl)aminophenol **S1** (420 mg, 2.32 mmol, 1 equiv) and α-acetylbutryrolactone (275 µL, 2.55 mmol, 1.1 equiv) were added to 70% $H_2SO_{4(aq)}$ (11.6 mL, 151 mmol) in a 100 mL rbf at 0 °C, then the reaction was allowed to reach room temperature and stirred overnight covered in foil to exclude light (24 h). The reaction was cooled in an ice bath with stirring while ice-cold NaOH (42 mL, 302 mmol; 7.2 M_{aq}) was added slowly to neutralise the mixture while maintaining a temperature below 5 °C. The reaction was filtered by suction and washed with ice-cold deionised water (3 x 30 mL) then suctioned further to form a yellowish filter cake. The filter cake was suspended in acetonitrile (50 mL) in a beaker and sonicated to form a yellow solution over white crystals (sodium sulfate). The solution was decanted and the solid sonicated in further acetonitrile and decanted (2 x 25 mL), then the solutions were combined, filtered and concentrated *in vacuo* to obtain the product **S2** as a white solid (595 mg, 2.04 mmol, 88%).

TLC (SiO_2, 5% MeOH in DCM) R_f = 0.39. **IR** (cm^{-1}) 1712 (C=O), 1680 (C=O). **^{1}H NMR** (601 MHz, DMSO-*d*$_6$) δ 10.06 (s, 1H, N*H*), 7.69 (d, *J* = 8.8 Hz, 1H, Ar*H*), 7.52 (d, *J* = 2.2 Hz, 1H, Ar*H*), 7.38 (dd, *J* = 8.8, 2.2 Hz, 1H, Ar*H*), 4.16 (q, *J* = 7.1 Hz, 2H, C*H*$_2$), 3.51 (t, *J* = 6.9 Hz, 2H, C*H*$_2$), 2.72 (t, *J* = 6.9 Hz, 2H, C*H*$_2$), 2.39 (s, 3H, C*H*$_3$), 1.25 (t, *J* = 7.1 Hz, 3H, C*H*$_3$). **HRMS (MALDI)** (M = $C_{15}H_{17}NO_5$) calc. $[M+H]^+$ = 292.1180, found = 292.1177.

7-amino-3-hydroxyethyl-4-methylcoumain (AHMC) (**1**)

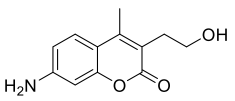

7-*N*-(Carboxyethyl)amino-3-hydroxyethyl-4-methylcoumarin **S2** (638 mg, 2.19 mmol, 1 equiv) and NaOH (1.05 g, 26.3 mmol, 12 equiv) was dissolved in water (6 mL) and stirred with heating (80 °C, 2 h). The reaction was cooled to room temperature and the pH neutralised by dropwise addition of conc. HCl (~2 mL) giving a thick cream-coloured precipitate. The precipitate was collected by suction filtration, washed with water and ice-cold acetonitrile, and dried under reduced pressure to give the product **1** as an off-white solid (477 mg, 2.18 mmol, 99%).

TLC (SiO_2, 5% MeOH in DCM) $R_f = 0.32$. **IR** (cm^{-1}) 1645 (C=O). **^{1}H NMR** (500 MHz, Methanol-d_4) δ 7.47 (d, *J* = 8.7 Hz, 1H, Ar*H*), 6.65 (dd, *J* = 8.7, 2.3 Hz, 1H, Ar*H*), 6.49 (d, *J* = 2.3 Hz, 1H, Ar*H*), 3.68 (t, *J* = 7.0 Hz, 2H, C*H_2*), 2.84 (t, *J* = 7.0 Hz, 2H, C*H_2*), 2.42 (s, 3H, C*H_3*). **^{13}C NMR** (126 MHz, Methanol-d_4) δ 164.95 (*C*=O), 155.66 (Ar*C*), 153.52 (Ar*C*), 151.73 (Ar*C*), 127.15 (Ar*C*H), 117.09 (Ar*C*), 113.18 (Ar*C*H), 112.05 (Ar*C*), 100.52 (Ar*C*H), 61.52 (*C*H_2), 31.61 (*C*H_2), 15.17 (*C*H_3). **HRMS (MALDI)** (M = $C_{12}H_{13}NO_3$) calc. $[M+H]^+$ = 220.0968, found = 220.0966.

HO-COLAZ-Phenol (**2**)

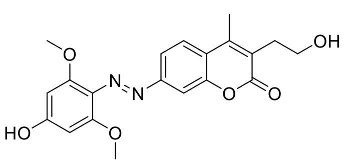

AHMC **1** (1.00 g, 4.56 mmol, 1 equiv) was suspended with stirring in aqueous hydrochloric acid (0.25 M, 57.0 mL, 3.1 equiv) in a 250 mL rbf and cooled in an ice bath. Sodium nitrite (627 mg, 9.12 mmol, 2 equiv) was dissolved in water (4.6 mL, 2 M) in a sample vial and cooled in an ice bath (15 minutes) then added in one portion to the AHMC suspension and stirred vigorously until the AHMC was dissolved (30 min). Aqueous sodium hydroxide (1 M, 4.80 mL, 4.84 mmol, 1.06 equiv) was added to a solution of 3,5-dimethoxyphenol (844 mg, 5.47 mmol, 1.2 equiv) and potassium carbonate (378 mg, 2.74 mmol, 0.6 equiv) in water (13 mL). This solution was added dropwise to the diazotised AHMC solution over 10 minutes, yielding a red precipitate, which was stirred for a further 15 minutes on ice. The solid was collected by suction filtration, washing with aqueous HCl (0.25 M, 40 mL). The wet solid was suspended in 200 mL boiling ethanol and filtered while hot. The filter cake was dried under vacuum to yield the product as an orange-brown solid (1.54 g, 4.01 mmol, 88%).

TLC (SiO_2, 5% MeOH in DCM) $R_f = 0.26$. **IR** (cm^{-1}) 1683 (C=O), 1615 (C=C), 1558 (N=N). **^{1}H NMR** (601 MHz, 323 K, DMSO-d_6) Mixture of isomers. Major *trans* isomer: δ 7.83 (d, *J* = 8.7 Hz, 1H, Ar*H*), 7.56 (dd, *J* = 8.7, 2.1 Hz, 1H, Ar*H*), 7.45 (d, *J* = 2.1 Hz, 1H, Ar*H*), 5.99 (d, *J* = 2.2 Hz, 1H, Ar*H*), 5.88 (d, *J* = 2.2 Hz, 1H, Ar*H*), 3.85 (s, 6H, C*H_3*), 3.56 (t, *J* = 6.9 Hz, 2H, C*H_2*), 2.78 (t, *J* = 6.9 Hz, 2H, C*H_2*), 2.45 (s, 3H, C*H_3*). **HRMS (MALDI)** (M = $C_{20}H_{20}N_2O_6$) calc. $[M+H]^+$ = 385.1394, found = 385.1393.

HO-COLAZ-OEt (**3**)

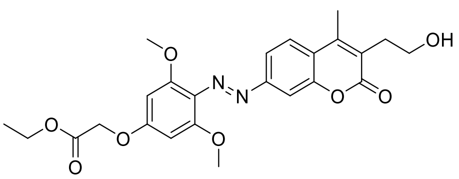

HO-COLAZ-Phenol **2** (1.50 g, 3.90 mmol, 1 equiv), potassium carbonate (1.08 g, 7.81 mmol, 2 equiv), and ethyl bromoacetate (476 µL, 4.29 mmol, 1.1 equiv) were suspended in DMF (35 mL) and heated to 80 °C with stirring (90 min), forming a deep red solution. The reaction was poured onto ethyl acetate (150 mL), washed with water (100 mL), saturated aqueous sodium hydrogen carbonate (3 x 50 mL), and brine (3 x 50 mL), then the organic layer was collected in a conical flask. An orange solid crystallised spontaneously from the deep red organic layer over 1 h, and the flask was placed in an ice bath for 1 h longer to encourage further crystallisation. The solid was collected by filtration and dried *in vacuo* yielding the first crop of product (1.20 g). The deep red solution was concentrated *in vacuo* and recrystallised from boiling ethyl acetate yield a second crop of product (205 mg). The aliquots were combined to yield the product **3** as an orange solid (1.40 g, 2.98 mmol, 76%).

TLC (SiO_2, 5% MeOH in DCM) R_f = 0.46; (SiO_2, 2:1 EtOAc : Petroleum ether) R_f = 0.28. **IR** (cm^{-1}) 1715 (br. C=O), 1598 (C=C), 1585 (C=C). **^{1}H NMR** (500 MHz, 298 K, Methanol-*d*$_4$) δ 7.91 (d, *J* = 8.6 Hz, 1H, Ar*H*), 7.78 (dd, *J* = 8.6, 2.0 Hz, 1H, Ar*H*), 7.63 (d, *J* = 2.0 Hz, 1H, Ar*H*), 6.40 (s, 2H, 2Ar*H*), 4.85 (s, 2H, CH_2), 4.29 (q, *J* = 7.1 Hz, 2H, CH_2), 3.89 (s, 6H, 2CH_3), 3.76 (t, *J* = 6.8 Hz, 2H, CH_2), 2.95 (t, *J* = 6.8 Hz, 2H, CH_2), 2.56 (s, 3H, CH_3), 1.32 (t, *J* = 7.1 Hz, 3H, CH_3). **^{13}C NMR** (126 MHz, 298K, Methanol-*d*$_4$) δ 170.38 (*C*=O), 163.58 (*C*=O), 163.38 (Ar*C*), 157.12 (2Ar*C*), 156.45 (Ar*C*), 153.98 (Ar*C*), 150.06 (Ar*C*), 128.75 (Ar*C*), 126.95 (Ar*C*H), 124.59 (Ar*C*), 122.94 (Ar*C*), 119.92 (Ar*C*H), 109.84 (Ar*C*H), 93.35 (2Ar*C*H), 66.39 (*C*H_2), 62.55 (*C*H_2), 61.26 (*C*H_2), 56.94 (2*C*H_3), 32.17 (*C*H_2), 15.57 (*C*H_3), 14.50 (*C*H_3). **HRMS (MALDI)** (M = $C_{24}H_{26}N_2O_8$) calc. $[M+H]^+$ = 471.1962, found = 471.1960.

Ts-COLAZ-OEt (**4**)

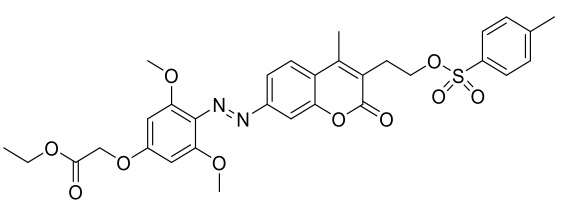

DMAP (36.0 mg, 298 umol, 0.1 equiv) was added to a suspension of HO-COLAZ-OEt **3** (1.40 g, 2.98 mmol, 1 equiv), DIPEA (1.59 mL, 8.93 mmol, 3 equiv) and tosyl chloride (852 mg, 4.47 mmol, 1.5 equiv) in anhydrous DCM (25 mL) and the reaction was stirred at reflux (55 °C, 16 h). Additional TsCl (568 mg, 2.98 mmol, 1 equiv) was added to the reaction and stirred at reflux further (55 °C, 30 h). The reaction was cooled to room temperature, and directly passed over a silica plug (ø 65 mm x 55 mm, 1-5% MeOH). The product-containing fractions were combined and concentrated *in vacuo* to obtain the product as a crispy dark red solid (1.632 g). This was purified by column chromatography (SiO_2, 50% ethyl acetate in petroleum ether) to yield product **4** as a red solid (1.04 g, 1.67 mmol, 56%).

TLC (SiO_2, 2:1 ethyl acetate : petroleum ether) R_f = 0.75. **IR** (cm^{-1}) 1753 (C=O), 1702 (C=O), 1595 (C=C), 1581 (C=C). **^{1}H NMR** (500 MHz, 353 K, DMSO-d_6) Mixture of isomers. Major *trans* isomer: δ 7.89 (d, *J* = 8.6 Hz, 1H, Ar*H*), 7.70 (dd, *J* = 8.6, 2.1 Hz, 1H, Ar*H*), 7.62 (d, *J* = 8.1 Hz, 2H, 2Ar*H*), 7.51 (d, *J* = 2.1 Hz, 1H), 7.23 (d, *J* = 8.1 Hz, 2H, 2Ar*H*), 6.43 (s, 2H, 2Ar*H*), 4.90 (s, 2H, CH_2), 4.32 (t, *J* = 6.2 Hz, 2H, CH_2), 4.24 (q, *J* = 7.1 Hz, 2H, CH_2), 3.84 (s, 6H, 2CH_3), 2.99 (t, *J* = 6.2 Hz, 2H, CH_2), 2.40 (s, 3H, CH_3), 2.27 (s, 3H, CH_3), 1.27 (t, *J* = 7.0 Hz, 3H, CH_3). **^{13}C NMR** (126 MHz, 353 K, DMSO-d_6) δ 168.25 (*C*=O), 161.12 (Ar*C*), 160.15 (*C*=O), 154.90 (2Ar*C*), 154.55 (Ar*C*), 152.18 (Ar*C*), 148.89 (Ar*C*), 144.70 (Ar*C*), 132.10 (Ar*C*), 129.81 (2Ar*C*H), 127.14 (2Ar*C*H), 127.07 (Ar*C*), 126.43 (Ar*C*H), 120.85 (Ar*C*), 120.82 (Ar*C*), 118.16 (Ar*C*H), 108.23 (Ar*C*H), 92.27 (2Ar*C*H), 68.95 (*C*H_2), 65.05 (*C*H_2), 60.76 (*C*H_2), 56.39 (2*C*H_3), 27.07 (*C*H_2), 20.94 (*C*H_3), 15.08 (*C*H_3), 14.08 (*C*H_3). **HRMS (MALDI)** (M = $C_{31}H_{32}N_2O_{10}S$) calc. $[M+H]^+$ = 625.1850, found = 625.1848.

N_3-COLAZ-OEt (**5**)

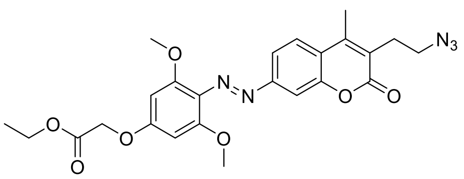

Sodium azide (268 mg, 4.12 mmol, 2.5 equiv) was added to a solution of Ts-COLAZ-OEt **4** (1.03 g, 1.65 mmol, 1 equiv) in dry DMF (16 mL) and heated with stirring in a sealed rbf (55 °C, 11 h). The reaction was poured onto ethyl acetate (150 mL) and washed with brine (2 x 50 mL), saturated aqueous sodium hydrogen carbonate (3 x 50 mL) and further brine (2 x 50 mL). The organic layer was dried over anhydrous magnesium sulfate, filtered and concentrated *in vacuo* yielding the product **5** as a red solid (708 mg, 1.43 mmol, 87%).

TLC (2:1 ethyl acetate : petroleum ether) R_f = 0.80. **IR** (cm^{-1}) 2091 (N=N=N), 1752 (C=O), 1704 (C=O), 1596 (C=C), 1578 (C=C). **^{1}H NMR** (500 MHz, 353 K, DMSO-d_6) δ 7.96 (d, *J* = 8.5 Hz, 1H, Ar*H*), 7.70 (dd, *J* = 8.5, 2.0 Hz, 1H, Ar*H*), 7.56 (d, *J* = 2.0 Hz, 1H, Ar*H*), 6.43 (s, 2H, 2Ar*H*), 4.90 (s, 2H, CH_2), 4.24 (q, *J* = 7.1 Hz, 2H, CH_2), 3.84 (s, 6H, 2CH_3), 3.55 (t, *J* = 7.0 Hz, 2H, CH_2), 2.96 (t, *J* = 7.0 Hz, 2H, CH_2), 2.53 (s, 3H, CH_3), 1.26 (t, *J* = 7.1 Hz, 3H, CH_3). **^{13}C NMR** (126 MHz, DMSO-d_6) δ 167.75 (*C*=O), 160.69 (Ar*C*), 160.02 (*C*=O), 154.58 (2Ar*C*), 154.38 (Ar*C*), 152.01 (Ar*C*), 147.90 (Ar*C*), 127.48 (Ar*C*), 125.92 (Ar*C*H), 122.19 (Ar*C*), 120.69 (Ar*C*), 117.76 (Ar*C*H), 108.01 (Ar*C*H), 92.77 (2Ar*C*H), 65.07 (*C*H_2), 60.32 (*C*H_2), 56.25 (2*C*H_3), 48.84 (*C*H_2), 26.78 (*C*H_2), 14.52 (*C*H_3), 13.61 (*C*H_3). **HRMS (MALDI)** (M = $C_{24}H_{25}N_5O_7$) calc. $[M+H]^+$ = 496.1827, found = 496.1824.

COLAZ linker (**6**)

Aqueous potassium hydroxide (1 M, 4.47 mL, 4.47 mmol, 2.5 equiv) was added to a solution

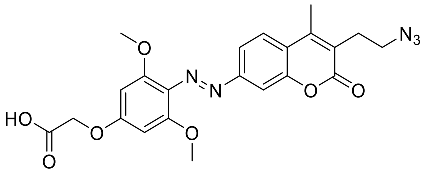

of N_3-COLAZ-OEt **5** (886 mg, 1.79 mmol, 1 equiv) in THF (15 mL) and stirred at room temperature (30 min). The reaction was poured onto aqueous HCl (40.0 mM, 6.00 mmol, 150 mL) giving a red precipitate which was extracted with DCM (3 x 50 mL). The organic layers were combined and dried over anhydrous magnesium sulfate, filtered and concentrated *in vacuo* to yield the product **6** as a red solid (822 mg, 1.76 mmol, 98%).

TLC (SiO_2, 10 % MeOH in DCM + acetic acid) R_f = 0.45. **IR** (cm^{-1}) 2085 (N=N=N), 1737 (C=O), 1702 (C=O), 1595 (C=C), 1572 (C=C). **^{1}H NMR** (500 MHz, 353 K, DMSO-d_6) δ 7.96 (d, *J* = 8.5 Hz, 1H, Ar*H*), 7.70 (dd, *J* = 8.5, 2.0 Hz, 1H, Ar*H*), 7.55 (d, *J* = 2.0 Hz, 1H, Ar*H*), 6.42 (s, 2H, 2Ar*H*), 4.80 (s, 2H, CH_2), 3.84 (s, 6H, 2CH_3), 3.55 (t, *J* = 7.0 Hz, 2H, CH_2), 2.96 (t, *J* = 7.0 Hz, 2H, CH_2), 2.53 (s, 3H, CH_3). **^{13}C NMR** (126 MHz, DMSO-d_6) δ 169.03 (*C*=O), 160.93 (Ar*C*), 160.03 (*C*=O), 154.64 (2Ar*C*), 154.43 (Ar*C*), 152.02 (Ar*C*), 147.91 (Ar*C*), 127.40 (Ar*C*), 125.92 (Ar*C*H), 122.17 (Ar*C*), 120.65 (Ar*C*), 117.77 (Ar*C*H), 107.99 (Ar*C*H), 92.74 (2Ar*C*H), 64.90 (*C*H_2), 56.24 (2*C*H_3), 48.85 (*C*H_2), 26.78 (*C*H_2), 14.52 (*C*H_3). **HRMS (MALDI)** (M = $C_{22}H_{21}N_5O_7$) calc. $[M+H]^+$ = 468.1514, found = 468.1511.

Fluorescence Excitation / Emission Spectra of COLAZ linker (**6**) and cleavage products (**S3**)

COLAZ linker **6** (1.0 mg) was dissolved at 0.5 mM concentration in either 0.25 M sodium dithionite solution in 1:1 *t*BuOH:water (cleavage solution) or 1:1 *t*BuOH:water (control), and allowed to stand at room temperature for 10 min. Aliquots of each solution (200 µL) were transferred to a 96-well plate, then fluorescence excitation and emission spectra were recorded on a ClarioStar Plus fluorescence plate reader. Excitation spectra were collected with top-optics, excitation wavelength (320 → 412 nm, bandwidth 10 nm), emission collected (479.2 nm, bandwidth 94 nm). Emission spectra were collected with top-optics, excitation wavelength (365 nm, bandwidth 16 nm), emission collected (400 – 500 nm, bandwidth 10 nm).

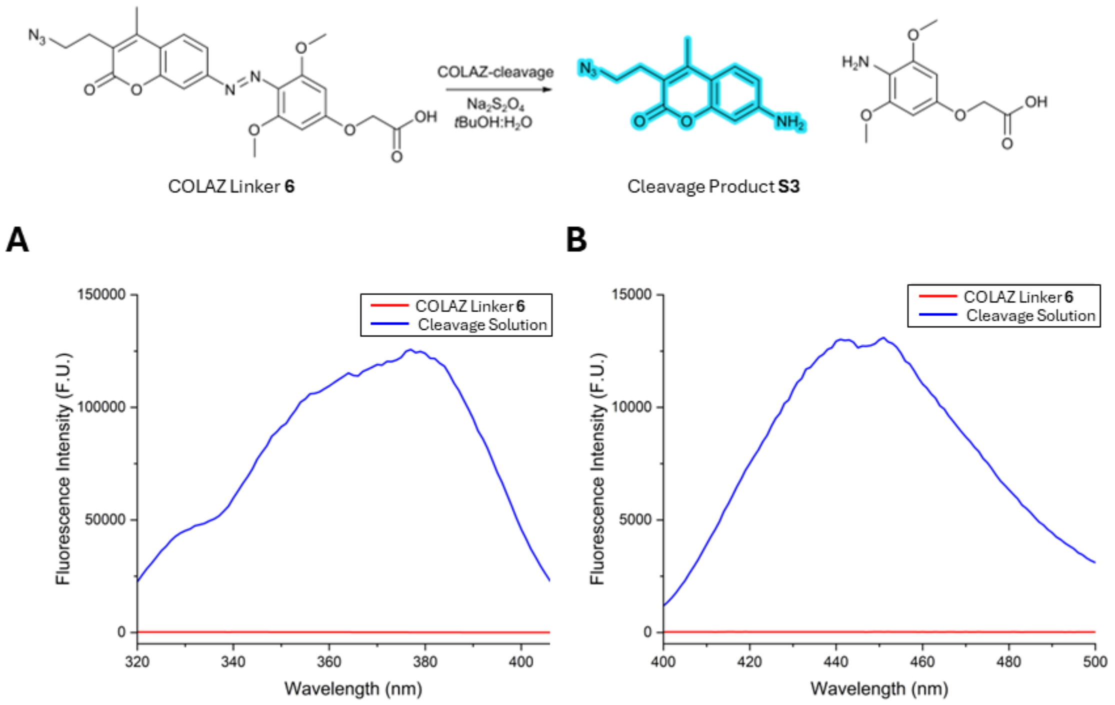

Figure S1. Fluorescence spectra for solutions of COLAZ linker **6** (1:1 *t*BuOH:water, control) and dithionite cleavage product **S3** (0.25 M sodium dithionite solution in 1:1 *t*BuOH:water, cleaved). (**A**) Excitation and (**B**) emission spectra.

NMR Spectra:

3-*N*-Carboxyethyl-aminophenol (**S1**) (^{1}H, 298 K, Methanol-d_4; ^{13}C, 298 K, MeOH-d_4)

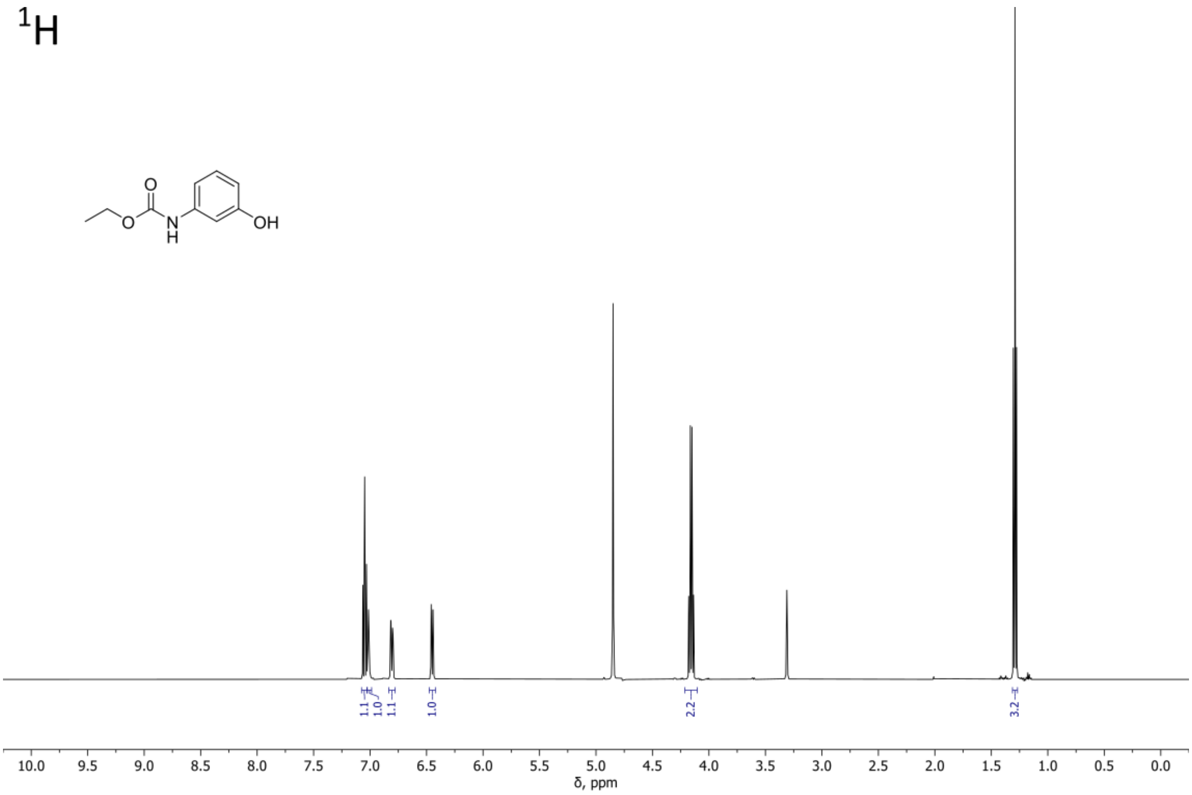

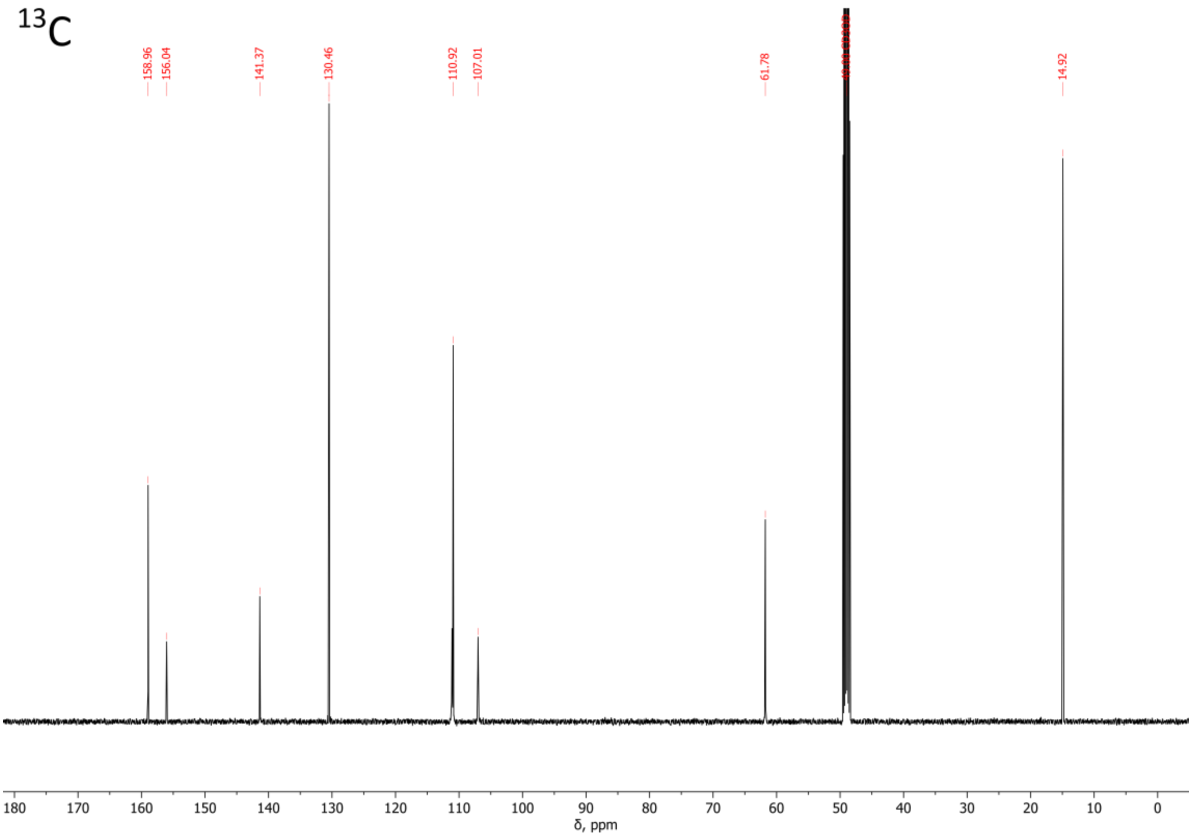

7-(*N*-Carboxyethyl)amino-3-hydroxyethyl-4-methylcoumarin (**S2**)

(^{1}H, 298 K, DMSO-d_6)

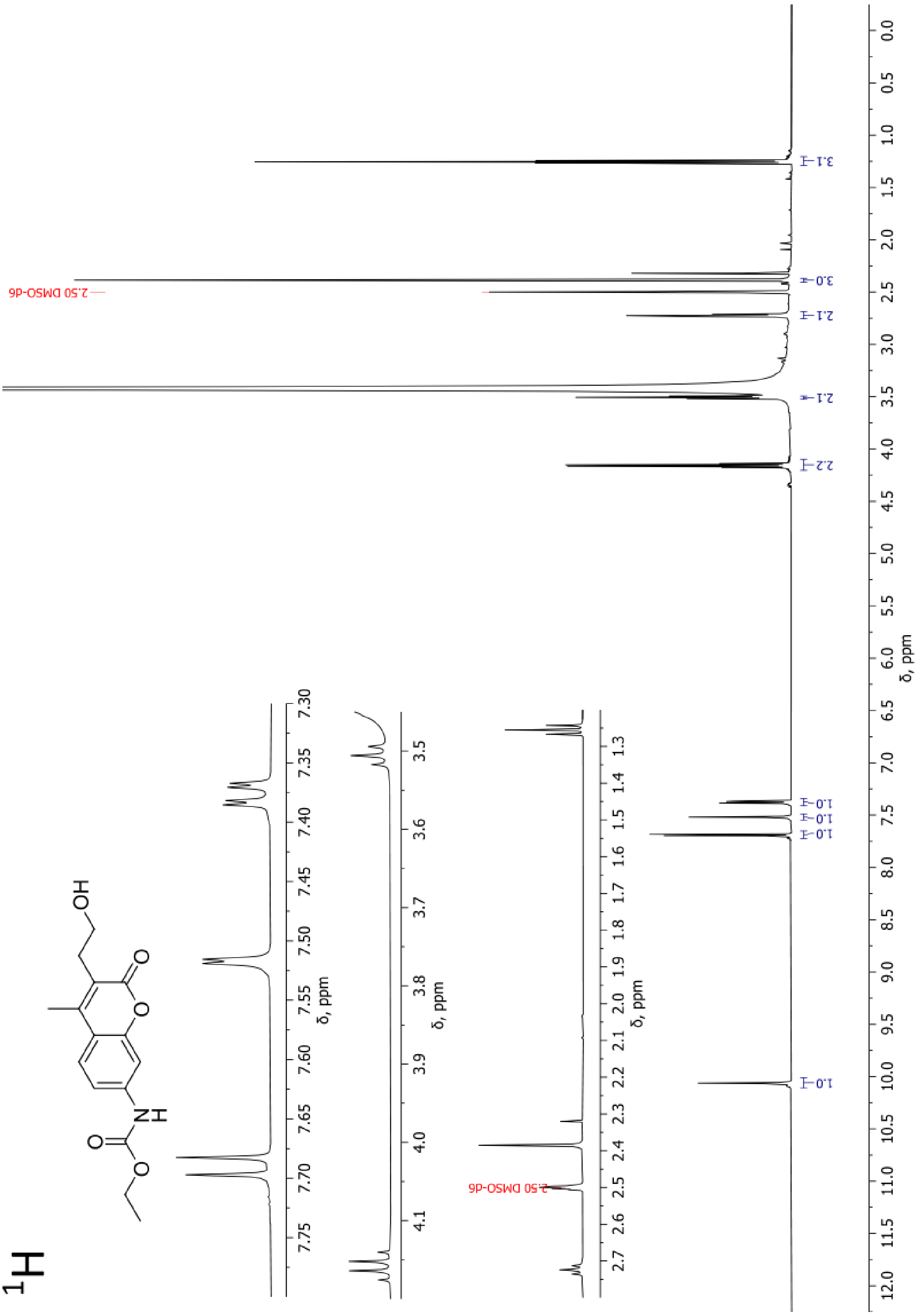

7-Amino-3-hydroxyethyl-4-methylcoumarin (AHMC) (**1**)

(^{1}H, 298 K, Methanol-d_4; ^{13}C, 298 K, Methanol-d_4)

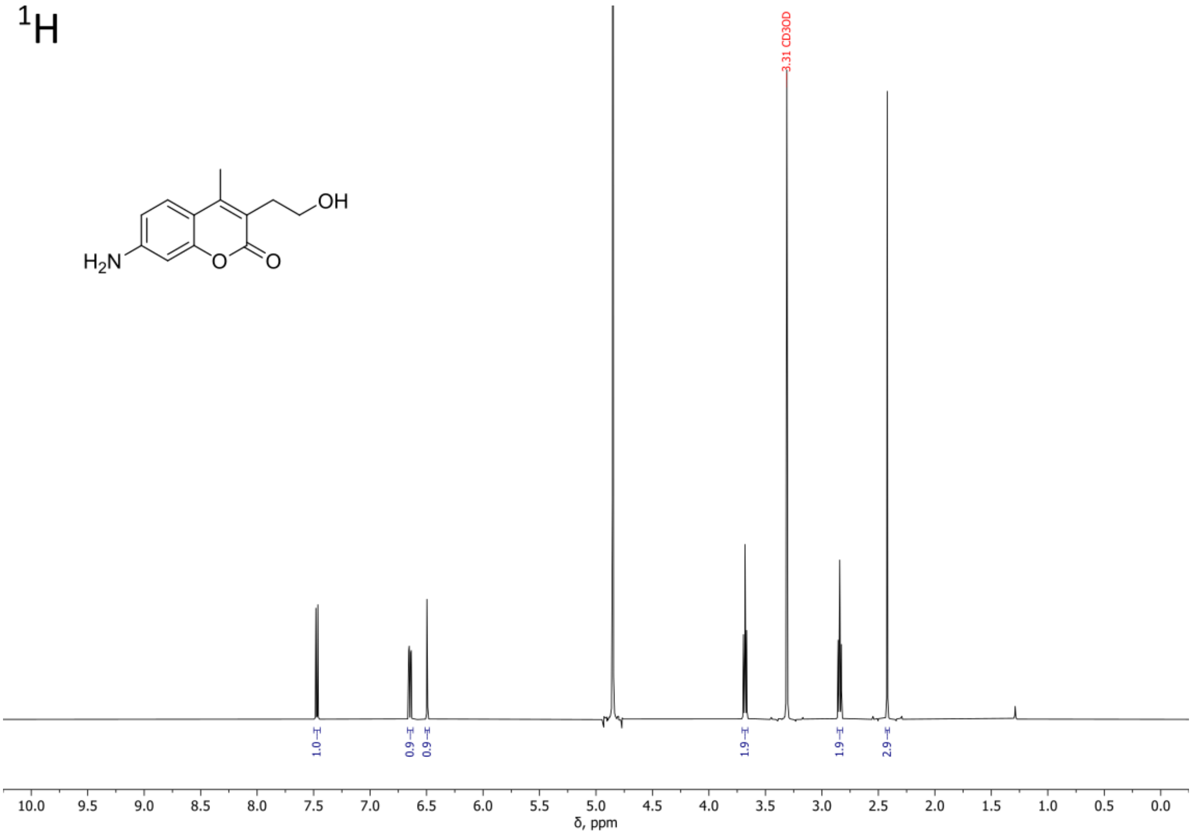

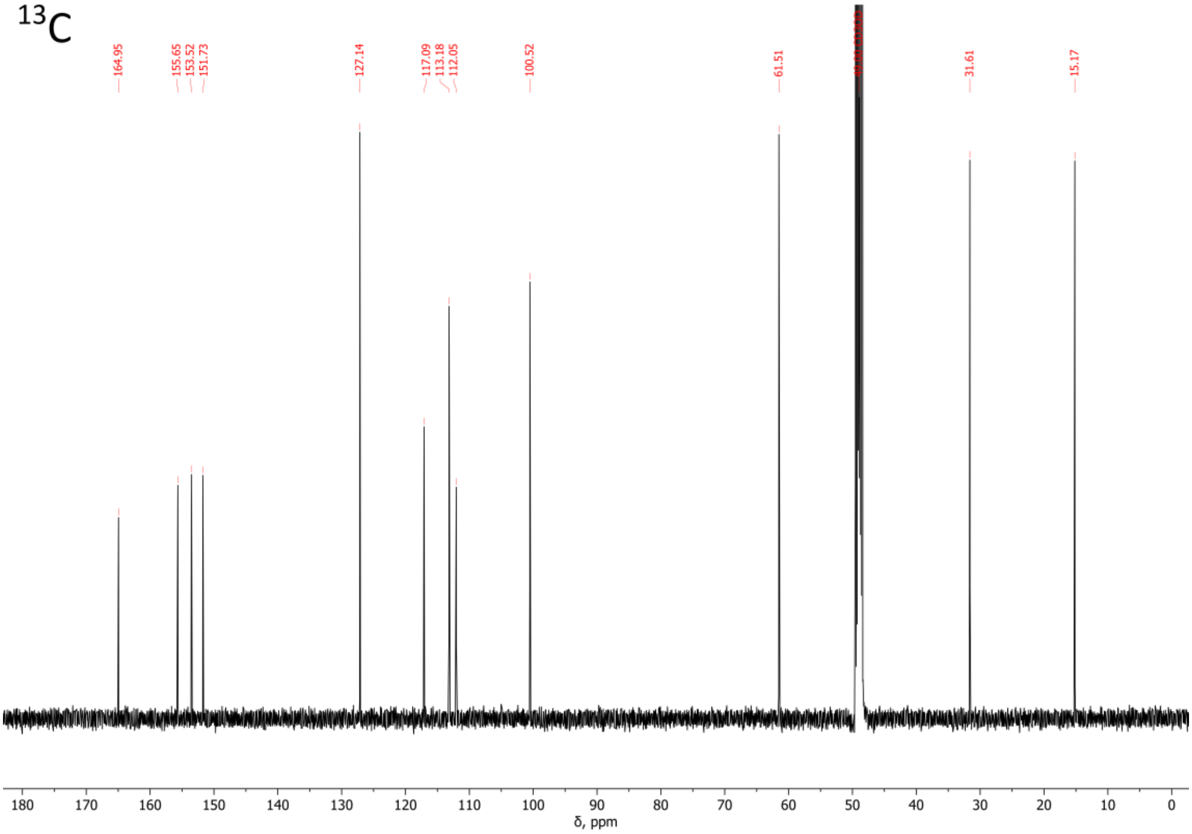

HO-COLAZ-Phenol (**2**)

(^{1}H, 323 K, DMSO-d_6)

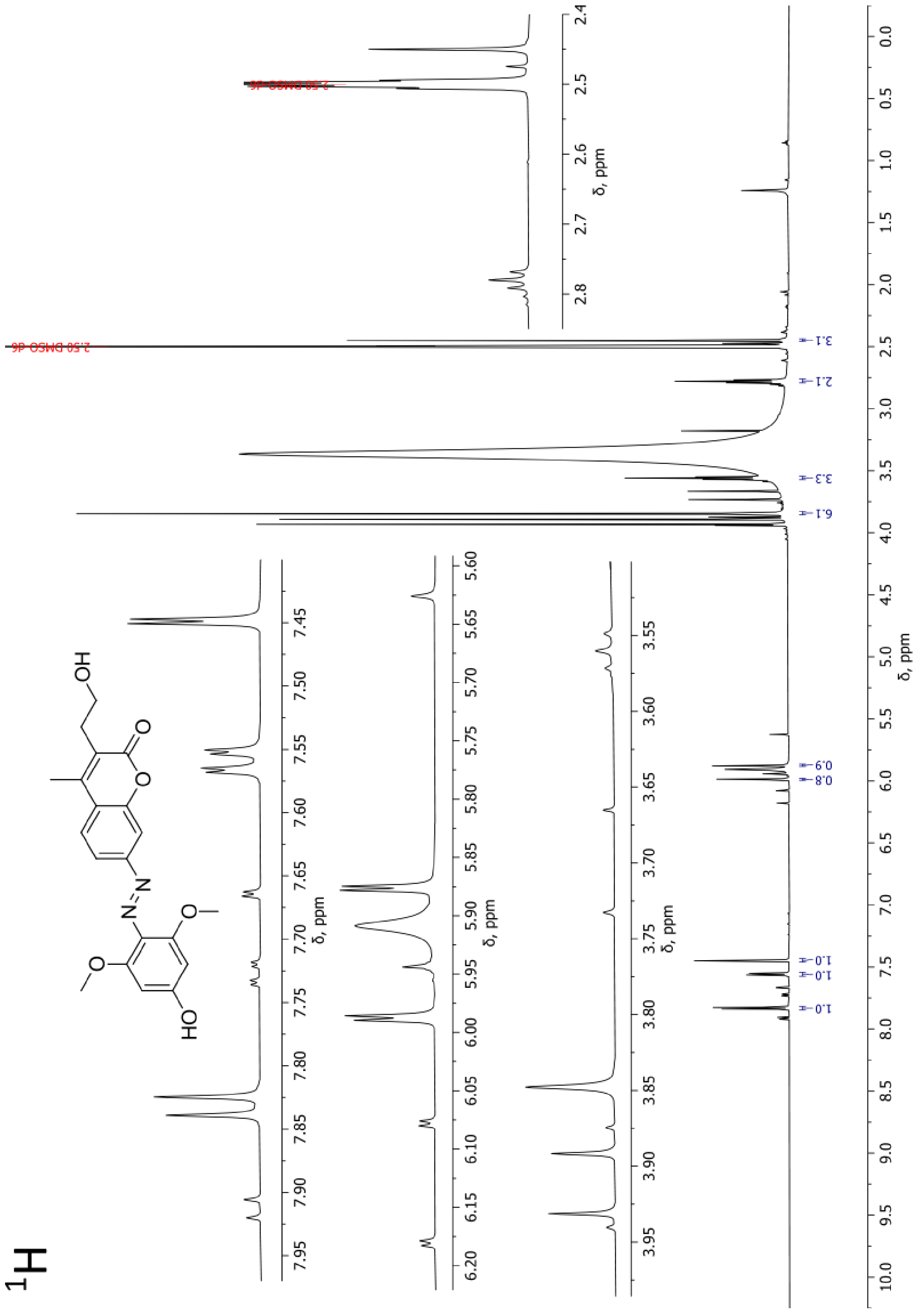

HO-COLAZ-OEt (**3**)

(^{1}H, 298 K, Methanol-d_4; ^{13}C, 298 K, Methanol-d_4)

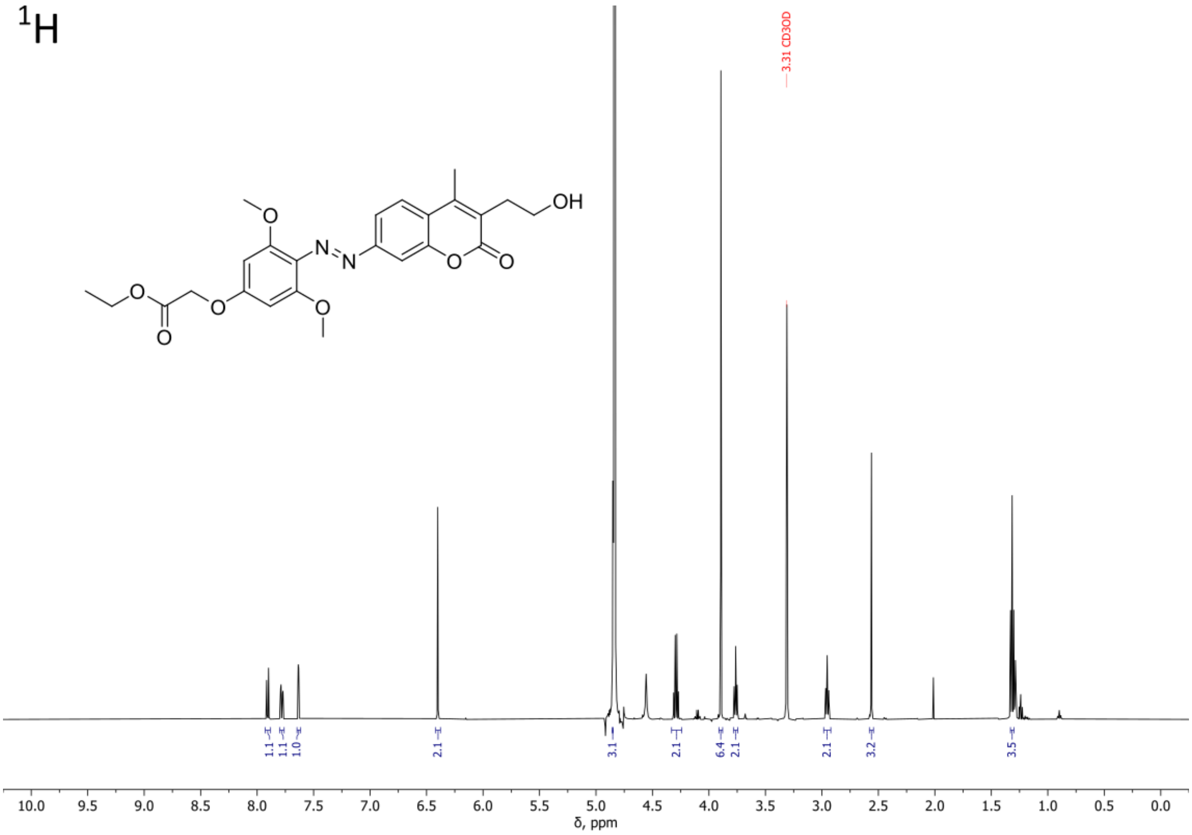

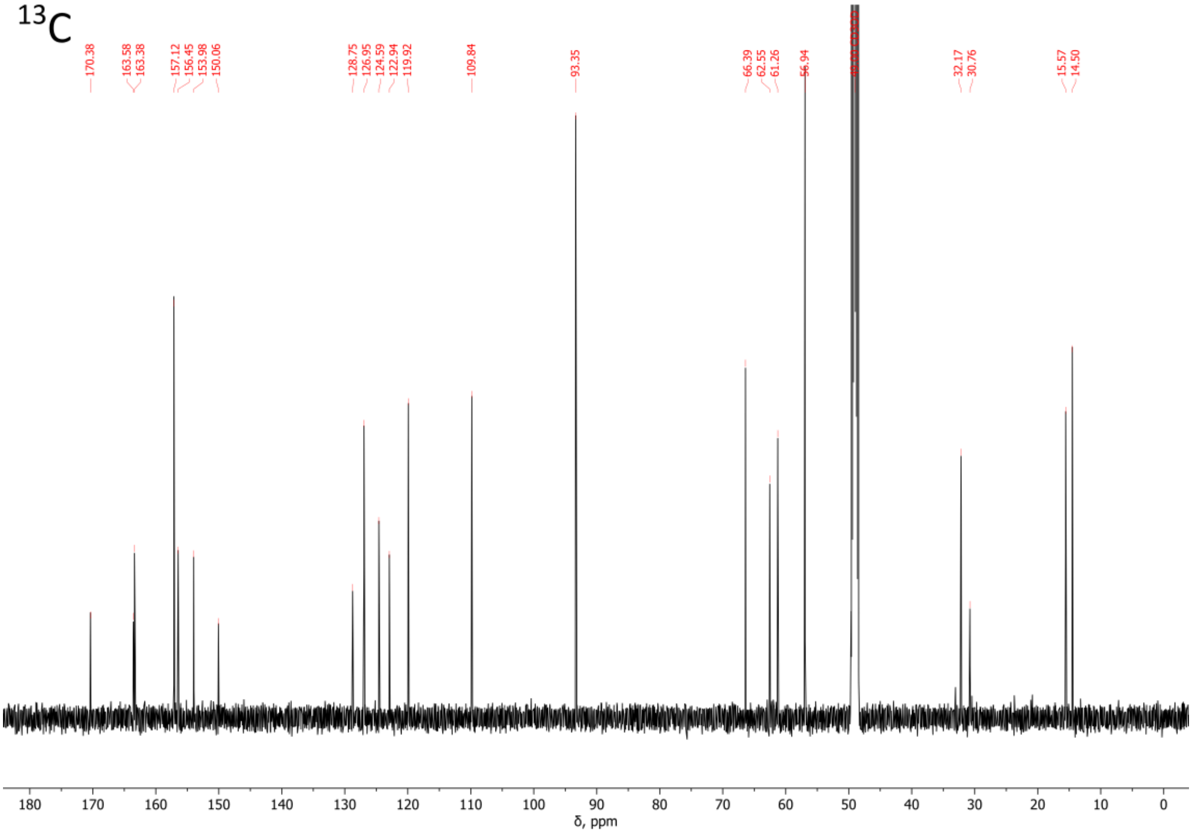

Ts-COLAZ-OEt (**4**)

(^{1}H, 353 K, DMSO-d_6; ^{13}C, 353 K, DMSO-d_6)

^{1}H

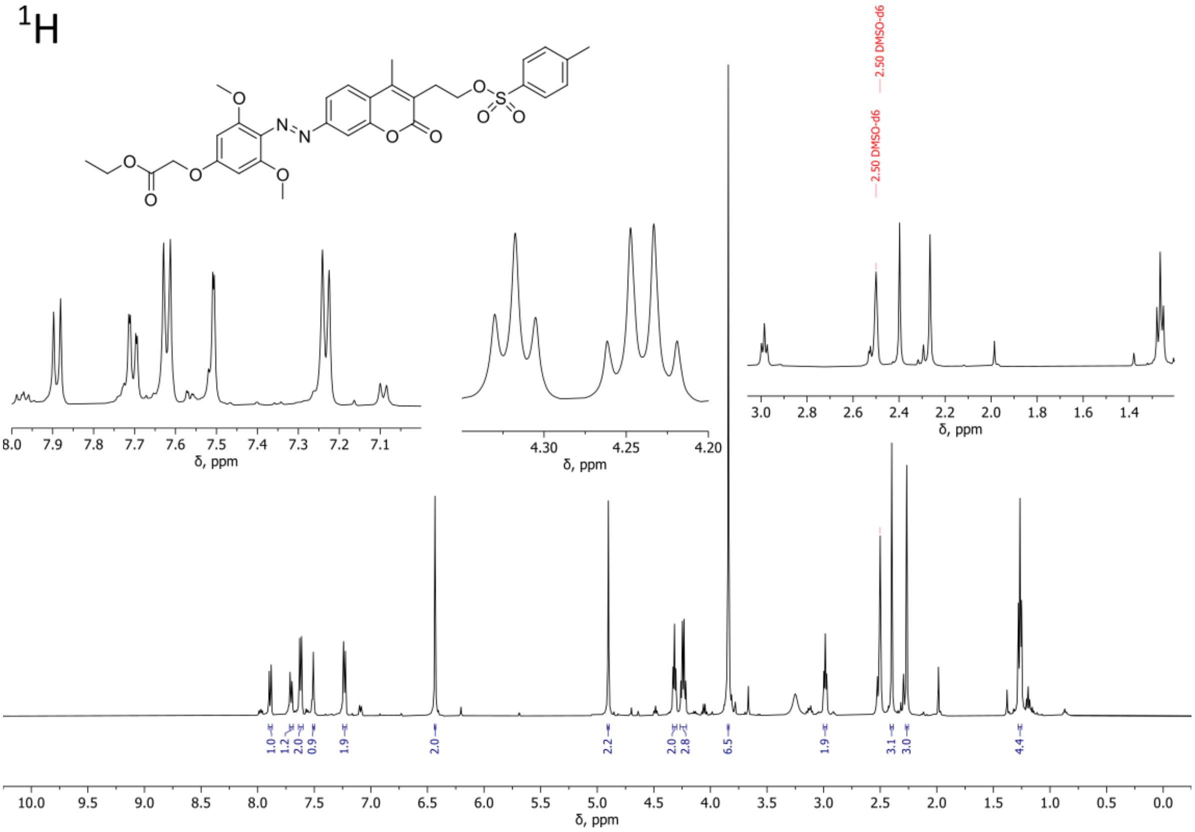

^{13}C

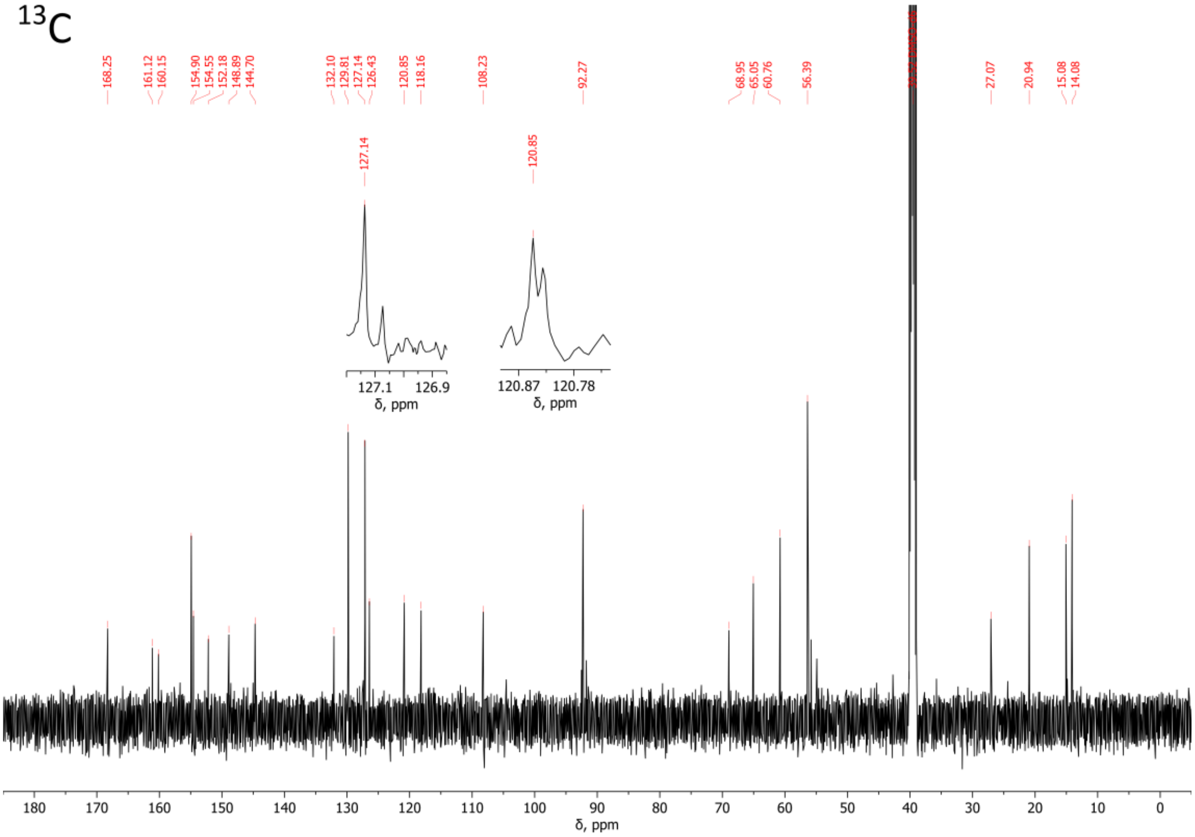

N_3-COLAZ-OEt (**5**)

(^1H, 353 K, DMSO-d_6; ^{13}C, 353 K, DMSO-d_6)

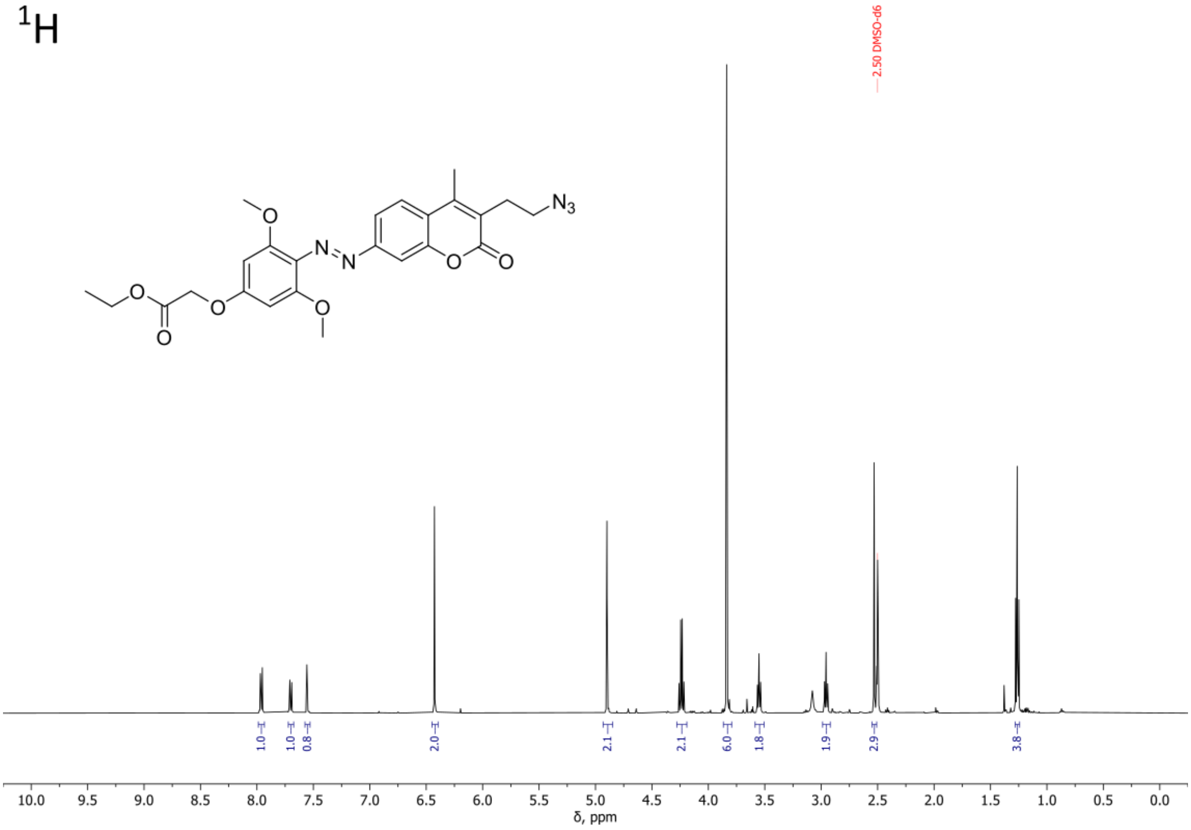

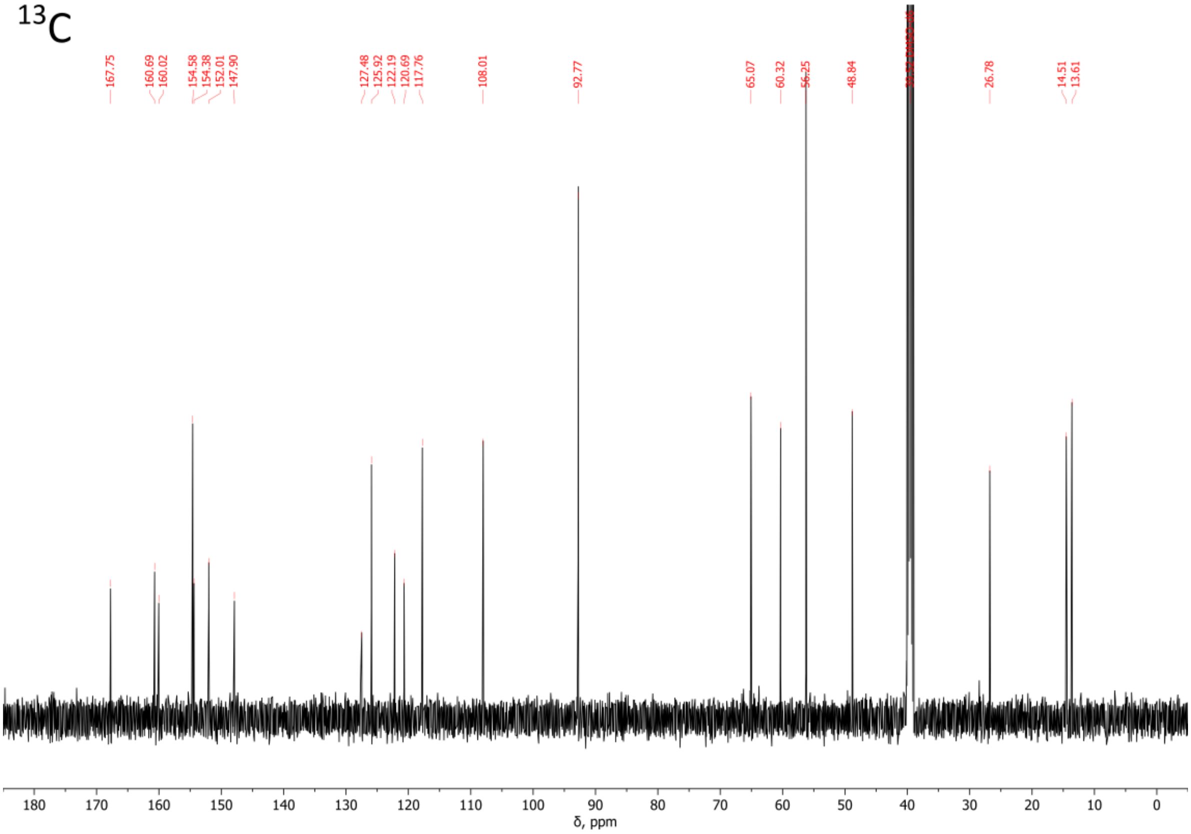

COLAZ linker (**6**)

(^{1}H, 353 K, DMSO-d_6; ^{13}C, 353 K, DMSO-d_6)

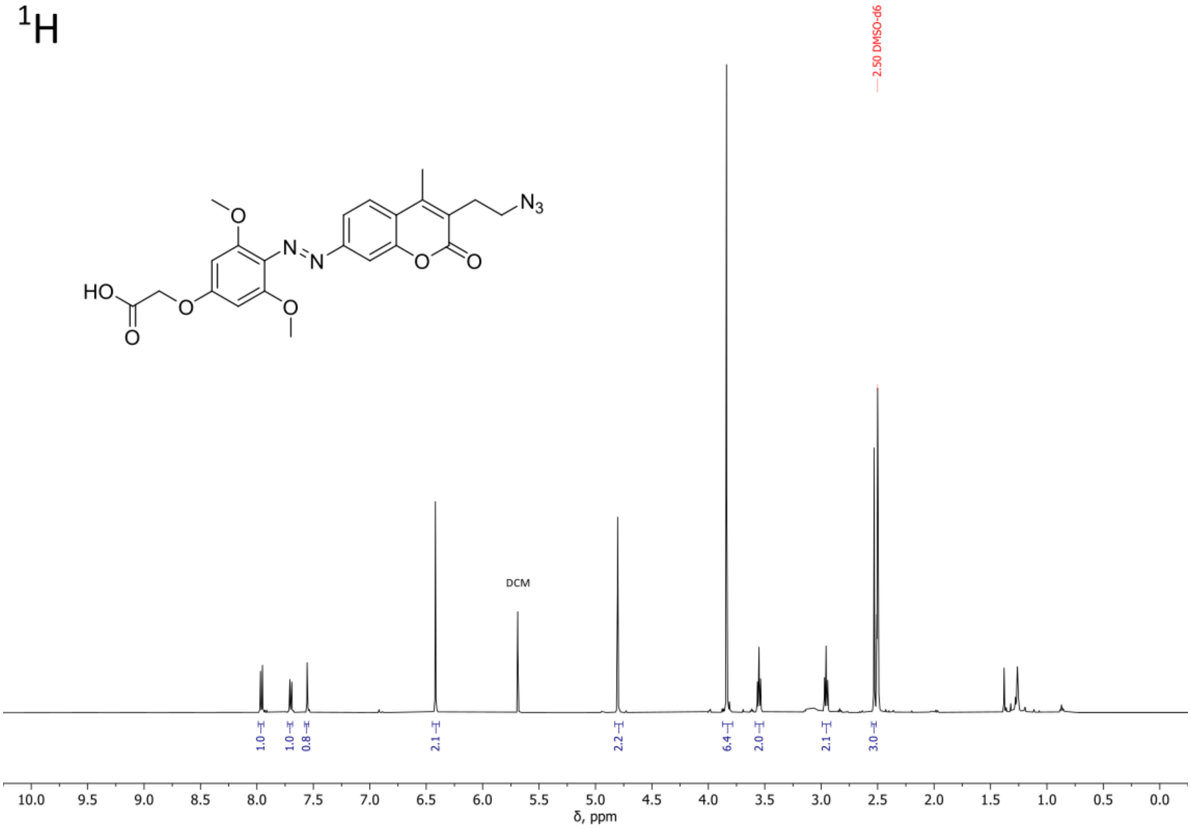

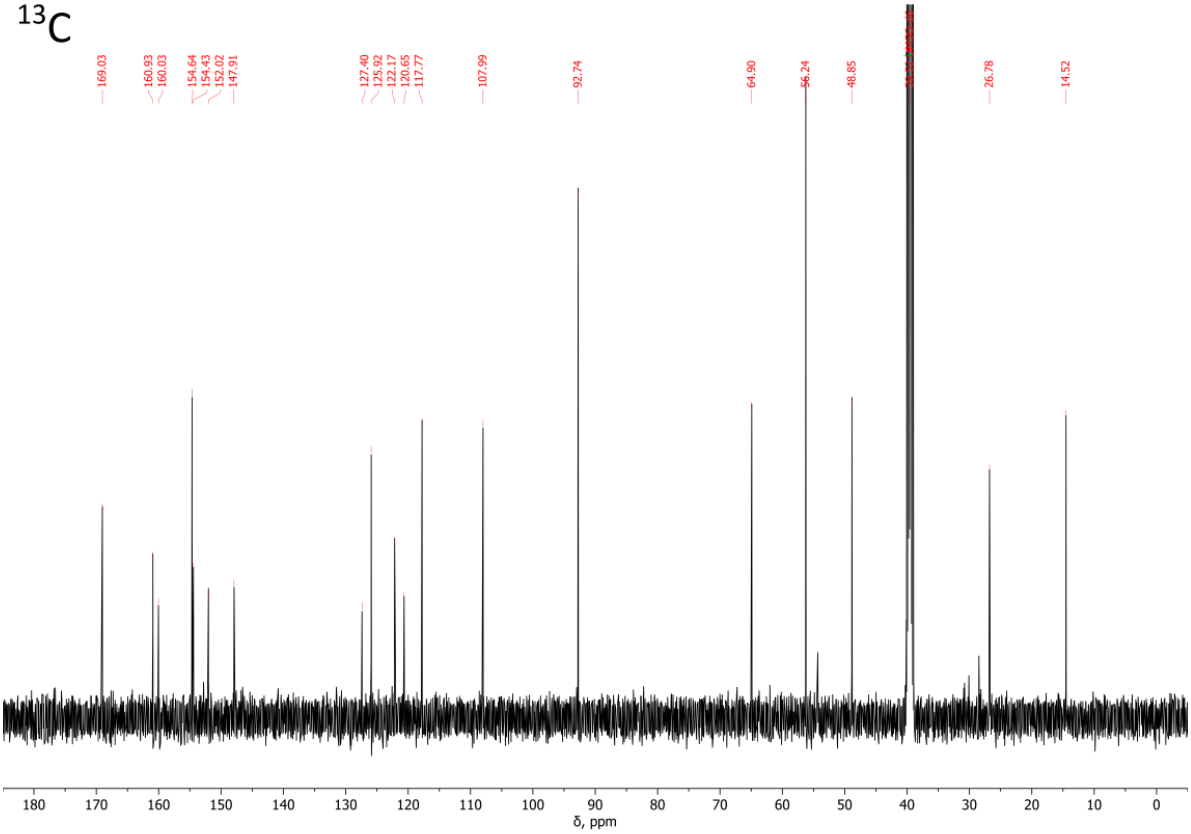

COLAZ Peptide Synthesis:

General Considerations:

Starting materials and reagents were obtained from commercial suppliers and used without further purification. Solutions of inorganic salts were freshly made for work-up procedures. COLAZ-loaded resins were prepared in a fume hood with manual synthesis steps performed on a benchtop rotary shaker by suspending the resins in syringes fitted with PET frits and luer lock valves, and draining solutions under vacuum.

Automated synthesis was performed on a Liberty blue™ peptide synthesiser. Amino acid coupling reactions were performed employing the required Fmoc-amino acids (0.2 M in DMF, 5 eq.) with DIC (0.5 M in DMF; 5 eq.) and Oxyma Pure™ (1 M in DMF, 5 eq.), and Fmoc deprotection was performed with morpholine (20% v/v in DMF, 4 mL). The WHISKEY peptides **Peptide 1** and **Peptide 2** were synthesised using standard coupling conditions (2.5 min, 90 °C), the R10 peptide - **Peptide 3** - was synthesised using arginine double coupling conditions (10 min, 50 °C). After completion, the solution was drained, washed with DMF (4 mL, 3 x 2 min), DCM (4 mL, 3 x 2 min), then Et_2O (4 mL, 3 x 2 min), and dried under vacuum. Peptides were then manually acetylated using acetic anhydride (20 % in DMF, 4 mL / 175 mg resin, 2 x 30 min) and washed.

Peptide purification was achieved on a Teledyne ACCQPrep HP150 prep-HPLC equipped with a RediSep Prep column (C18, 100Å, 5 µm x 20 µm x 150 µm). The total run time was 20 min at a flow rate of 18.9 mL min^{-1}. A binary solvent system was used; A = H_2O + 0.1% TFA and B = MeCN + 0.1% TFA. For WHISKEY peptides (Prep method A), the elution program was isocratic for 2 min (90A : 10B) then a linear gradient from 2 min (80A : 20B) to 15.0 min (60A : 40B). After 10 mins, the ratio was kept isocratic for 5 mins at (0A : 100B) to ensure full elution of loaded material. For R10 peptides (Prep method B), the elution program was isocratic for 2 min (90A : 10B) then a linear gradient from 2 min (80A : 20B) to 15.0 min (75A : 25B). After 10 mins, the ratio was kept isocratic for 5 mins at (0A : 100B) to ensure full elution of loaded material. The gradient is automatically kept at isocratic when an absorbance peak is detected.

UPLC data was recorded on a Shimadzu Nexera instrument. The UPLC method used a ACQUITY UPLC BEH C18 (1.7 Å, 2.1 x 100 mm). The total run time was 15.0 min at a flow rate of 1.0 mL min^{-1} and the column was maintained at 30 ± 1 °C. A binary solvent system was used; A = H_2O + 0.1% TFA and B = MeCN + 0.1% TFA. The elution program was isocratic for 2.0 min (95A : 5B) then a linear gradient from 2.1 min (80A : 20B) to 9.0 min (50A : 50B). After 9.1 mins, the gradient was kept at isocratic at (0A : 100B) for 4.9 min before recovery of the initial conditions over 0.1 min and equilibration over 1.0 min.

High resolution mass spectrometry (HRMS) analysis was performed via the School of Chemistry's MS facility using matrix assisted laser desorption ionisation (MALDI). Samples were run using a 12T SolariX FT-ICR MS equipped with MALDI ionization using a SmartBeam II UV laser (Bruker Daltonics, Bremen, Germany) in positive ion mode. Samples were co-crystalised on a Bruker MTP 384 ground steel plate with CHCA (10 mg/mL). A m/z range of 150 – 3000 was acquired at 30 % laser power.

LC-MS samples were analysed on an Agilent 6230 LC / TOF walk-up system at the school of chemistry's MS facility. The HPLC method used a Zorbax extend-C18 Column (1.8 Å, 2.1 x 50 mm). The total run time was 9.0 min at a flow rate of 500 µL min^{-1} and the column was maintained at 45 ± 1 °C. A binary solvent system was used; A = H_2O + 0.1% FA and B = MeCN + 0.1% FA. The elution program was isocratic for 0.5 min (95A : 5B) then a linear gradient from 0.5 min to 5.0 min (5A : 95B). The ratio was kept at isocratic until 6 mins (5A : 95B) before recovery of the initial conditions over 0.1 min and equilibration over 2.9 mins.

Micro-cleavage loading determination was performed on ClarioStar Plus fluorescence plate reader using top-optics laser (λ_{ex} = 380 nm, λ_{em} = 445 nm). Fmoc-deprotection loading determination was performed on a Cole-Parmer SP-200-UV Benchtop UV/Visible Spectrophotometer (λ = 301 nm) with quartz cuvette (*l* = 1 cm).

Synthesis:

<u>Loading of TentaGel S NH_2 resin with COLAZ linker</u>

A microwave tube was charged with TentaGel® S NH_2 resin (175 mg, 49.0 µmol, assumed at manufacture's loading 0.28 mmol/g). A solution of COLAZ linker **6** (69.3 mg, 148 µmol, 3 equiv), Oxyma pure (21.0 mg, 148 µmol, 3 equiv) and DIC (22.9 µl, 148 µmol, 3 equiv) in DMF (10 mL, 15 mM of **6**) was added and subjected to microwave radiation (70 °C, 30 min + 10 min). The solution was drained into a syringe vessel equipped with a PET frit, washed with DMF (4 mL, 3 x 2 min), DCM (4 mL, 3 x 2 min), then Et_2O (4 mL, 3 x 2 min), and dried under vacuum to yield **COLAZ resin**.

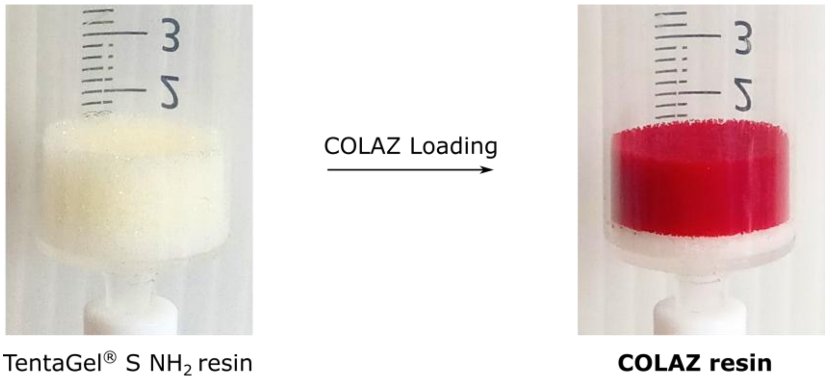

Figure S2. Colouration of TentaGel® resin on loading with the COLAZ linker **6**.

Loading of Rink AM Resin with COLAZ linker

A microwave tube was charged with Rink-Amide AM resin (63 mg, 50 µmol, assumed at manufacturer's loading 0.80 mmol/g). A solution of COLAZ linker **6** (70.0 mg, 150 µmol, 3 equiv), Oxyma pure (21.0 mg, 150 µmol, 3 equiv) and DIC (23.0 µl, 150 µmol, 3 equiv) in DMF (10 mL, 15 mM to **6**) was added and subjected to microwave radiation (70 °C, 30 min + 10 min). The solution was drained into a syringe vessel equipped with a PET frit, washed with DMF (4 mL, 3 x 2 min), DCM (4 mL, 3 x 2 min), then Et_2O (4 mL, 3 x 2 min), and dried under vacuum to yield **COLAZ-Rink AM resin**.

Solid-Phase CuAAC Conjugation

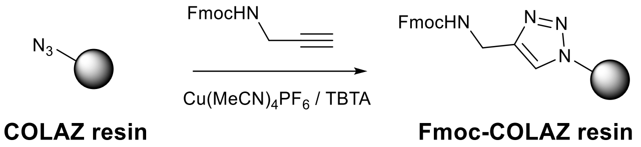

COLAZ resin (TentaGel) or **COLAZ-Rink AM resin** (50 µmol, assumed at manufacture's loading) was swollen in THF (4 mL, 20 min, then drained. A solution of *N*-Fmoc-propargylamine (27.7 mg, 100 µmol, 2.0 equiv) in THF (2 mL) was added, followed by a solution of $Cu(MeCN)_4PF_6$ (18.7 mg, 50.0 µmol, 1.0 equiv) and TBTA (26.5 mg, 50.0 µmol, 1.0 equiv) in THF (2 mL, 26 mM to the alkyne), and the reaction shaken at room temperature (2 h). The solution was drained, washed with DMF (4 mL, 3 x 2 min), DCM (4 mL, 3 x 2 min), then Et_2O (4 mL, 3 x 2 min), and dried under vacuum to yield **Fmoc-COLAZ resin** or **Fmoc-COLAZ-Rink AM resin**.

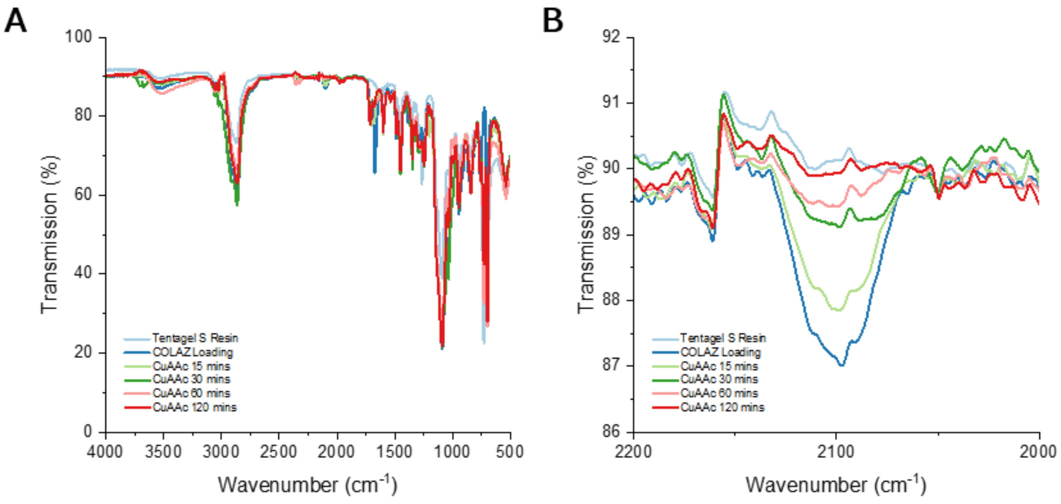

Figure S3. Tracking on-resin click coupling using FT-ATR-IR. (**A**) Full spectra overlaid (0-120 min), and (**B**) Zoom to track the disappearance of the azide peak of **COLAZ resin** at ~2100 cm^{-1} during click coupling to produce **Fmoc-COLAZ resin**.

Micro-cleavage of COLAZ resins for LC-MS analysis

A micro-spatula aliquot of COLAZ-functionalised resin was placed in an Eppendorf tube and soaked in 1:1 *t*BuOH:water. A micro-spatula of sodium dithionite was added and the solution was shaken at room temperature until complete decolourisation of the resin was observed (5-10 min), then the solution was diluted or extracted as desired for analysis.

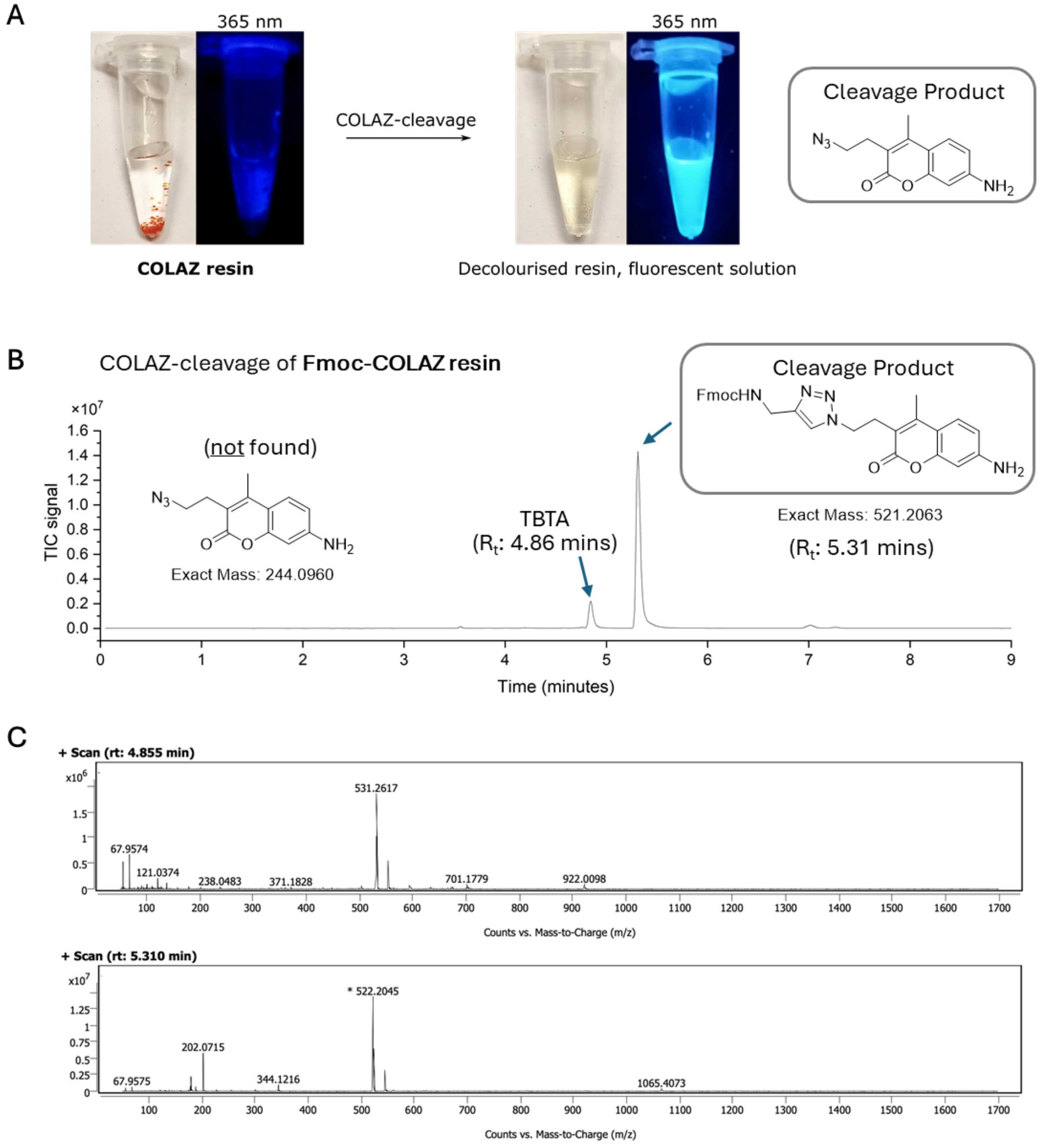

Figure S4. Micro-cleavage of COLAZ-functionalised resins. (**A**) Cleavage of **COLAZ resin** showing the characteristic resin decolourisation and fluorescence of the resulting solution containing AMC derivative **S3**. (**B**) and (**C**) Micro-cleavage of **Fmoc-COLAZ resin** confirming CuAAC reaction completion after 2 h: (**B**) LC-MS TIC chromatogram; and (**C**) MS data for peaks at 4.86 and 5.31 mins.

Loading Determination by COLAZ cleavage

COLAZ resin (25 mg) was cleaved using 0.25 M sodium dithionite solution in 1:1 *t*BuOH:water (4 x 4 mL), and the combined eluents were made up to 25 mL in a volumetric flask with 1:1 mixture of *t*BuOH:water. The fluorescence intensity of a 10X diluted sample was measured on a ClarioStar Plus fluorescence plate reader (λ_{ex} = 380 nm, λ_{em} = 445 nm). The value was referenced to a calibration curve made using a dilution series of a desalted and lyophilised isolated sample of cleavage product **S3**.

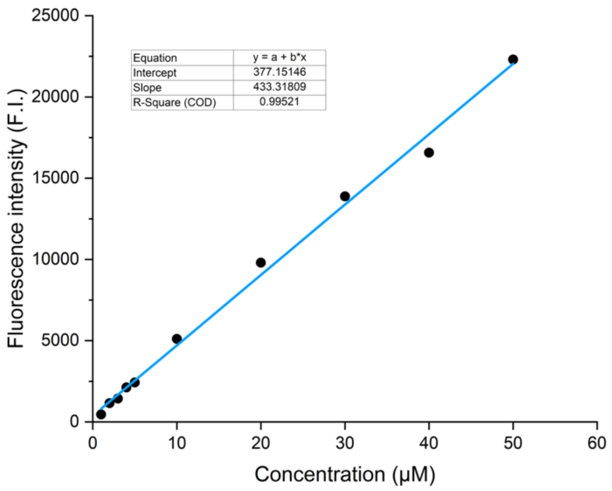

Figure S5. Calibration curve of an isolated sample of **COLAZ resin** cleavage product **S3** used to quantify COLAZ linker loading onto TentaGel® resin.

Exemplar calculation:

Calibration Curve: $y = 377.15x + 433.32$

Measured fluorescence Intensity from 25 mg resin cleavage = 8464 (F.U.)

Concentration of undiluted cleavage sample: $c = \frac{8464-433.32}{377.15} \times 10 = 213\ \mu\text{M}$

Moles from 25 mg resin cleavage sample: $n = cV = 213\ \mu M\ \times\ 0.025\ L = 5.32\ \mu\text{mol}$

Actual loading per grams of resin: $5.32\ \mu mol \div 0.025\ g = 213\ \mu mol/g\ \approx 0.21\ \text{mmol}$

% loading = $\frac{Actual\ loading\ (mmol)}{Manufacturer's\ loading\ (mmol)} \times\ 100 = \frac{0.21\ mmol}{0.28\ mmol} \times 100\ = 75\ \%$

Loading Determination using Fmoc deprotection

TentaGel-NH_2 resin (49.5 mg, stated loading 0.28 mmol g^{-1}) was loaded with COLAZ linker **6**, and the resulting **COLAZ resin** was subjected to the CuAAc click reaction protocol to form **Fmoc-COLAZ resin**. To ensure complete cleavage of the Fmoc group, the resin was subjected to 3 cycles of Fmoc deprotection solution (20% piperidine in DMF, 1 mL per cycle), each cycle lasting 10 minutes. The combined cleavage solutions (3 mL in total) were mixed thoroughly and an aliquot (50 µL) was diluted 100 times with 20% piperidine in DMF to maintain an absorbance reading less than 1.00. The absorbance of the fluorenylmethyl-piperidine adduct was measured at 301 nm, on a bench-top UV-Vis spectrometer.

N-(9*H*-Fluoren-9-ylmethyl)-piperidine:[1] $\varepsilon_{301\ nm}$ = 8021 L mol^{-1} cm^{-1}

The equivalent procedure was repeated with Rink-amide AM resin (22.7 mg, stated loading 0.80 mmol g^{-1}).

Exemplar calculations:

TentaGel resin (49.5 mg): Absorbance from the 100-fold diluted solution = 0.26

$$c = \frac{A}{\varepsilon l}$$

$$c = \frac{0.26}{8021} = 3.24 \times 10^{-5}\ \mathrm{M}$$

$$n = cV = 3.24 \times 10^{-5} \times 0.003 \times 100 = 9.72 \times 10^{-3}\ \mathrm{mmol}$$

$$Loading = 9.72 \times 10^{-3} \div 0.0495\ \mathrm{g} = 0.196\ \mathrm{mmol\ g^{-1}}$$

$$\%\ loading = \frac{0.196}{0.28} \times 100 \approx 70\ \%$$

Rink-Amide AM resin (22.7 mg): Absorbance from the 100-fold diluted solution = 0.33

$$c = \frac{A}{\varepsilon l}$$

$$c = \frac{0.33}{8021} = 4.11 \times 10^{-5}\ \mathrm{M}$$

$$n = cV = 4.11 \times 10^{-5} \times 0.003 \times 100 = 0.012\ \mathrm{mmol}$$

$$Loading = 0.012 \div 0.0227\ \mathrm{g} = 0.544\ \mathrm{mmol\ g^{-1}}$$

$$\%\ loading = \frac{0.54}{0.80} \times 100 \approx 68\ \%$$

[1] Eissler, S.; Kley, M.; Bächle, D.; Loidl, G.; Meier, T.; Samson, D. *J. Pept. Sci.*, 2017, **23**, 757–762.

Ac-COLAZ resin stability studies

Base stability:

Fmoc-COLAZ resin was Fmoc-deprotected, then acetylated with acetic anhydride (20% in DMF, 2 x 20 mins) and dried to yield **Ac-COLAZ resin**. A fritted syringe was charged with 25 mg of **Ac-COLAZ resin**, followed by swelling in DMF (10 minutes). The resin was then treated repeatedly with Fmoc deprotection solution (20 % morpholine in DMF, 1 mL, 15 x 10 min cycles). In between cycles, the resin was washed with DMF (3 × 1 mL), and a micro-aliquot of the resin (approximately 50 beads) was subjected to COLAZ-cleavage (0.25 M sodium dithionite solution in 1:1 *t*BuOH:water), and the eluent collected by centrifugation. The fluorescence of the eluent (containing cleaved coumarin species) was measured on a ClarioStar Plus fluorescence plate reader (λ_{ex} = 380 nm, λ_{em} = 445 nm).

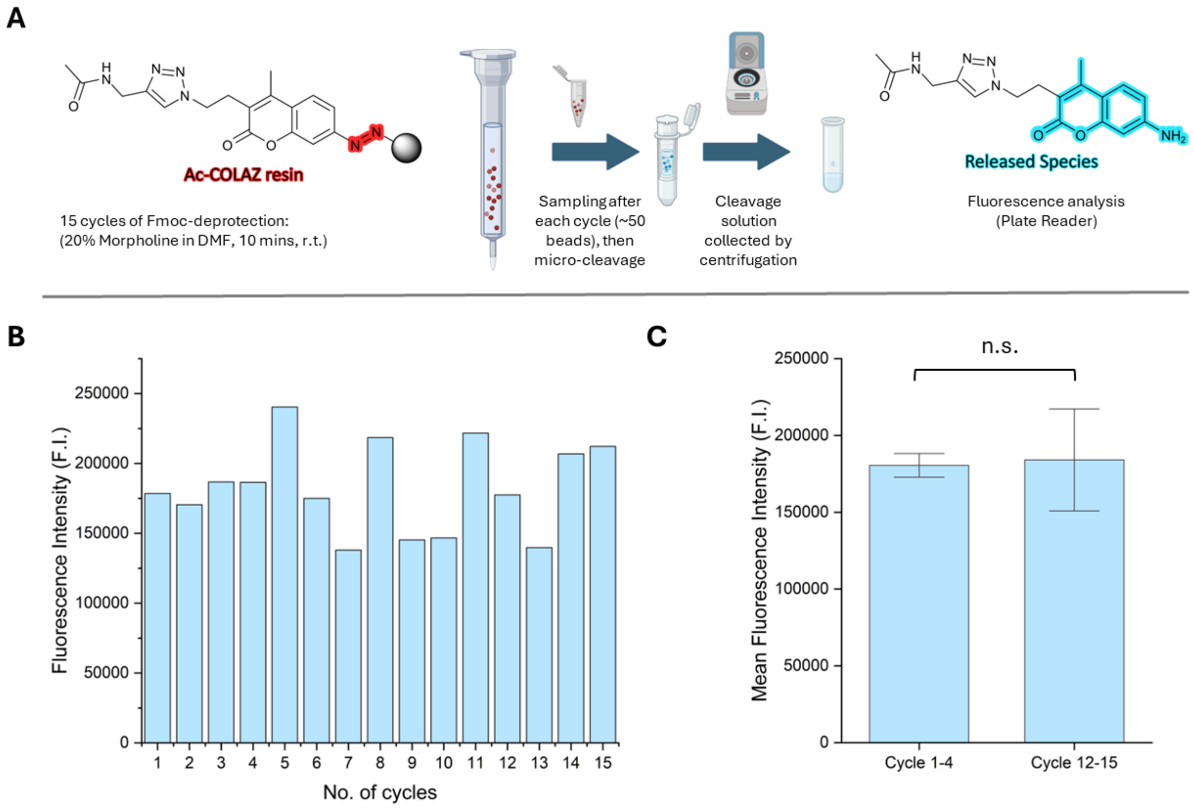

Figure S6. Base stability studies on **Ac-COLAZ resin**. (**A**) Schematic describing the workflow. (**B**) Bar chart showing the fluorescence intensity of the released coumarin for each deprotection cycle. (**C**) Comparison of the initial and final cycles presented as mean values with error bars representing ±1 standard deviation; statistical analysis was performed using a two-sample *t*-test assuming unequal variances, with $p > 0.05$.

Acid stability:

Fmoc-COLAZ resin was Fmoc-deprotected, then acetylated with acetic anhydride (20% in DMF, 2 x 20 mins) and dried to yield **Ac-COLAZ resin**. A fritted syringe was charged with 25 mg of **Ac-COLAZ resin**, followed by swelling in DMF (10 minutes). The resin was then treated repeatedly with side-chain deprotection cocktail (TFA : H_2O : TIPS 95 : 2.5 : 2.5, 1 mL, 7 x 1 h cycles). In between cycles, the resin was washed with DMF (3 × 1 mL), and a micro-aliquot of the resin (approximately 50 beads) was subjected to COLAZ-cleavage (0.25 M sodium dithionite solution in 1:1 *t*BuOH:water), and the eluent collected by centrifugation. The fluorescence of the eluent (containing cleaved coumarin species) was measured on a ClarioStar Plus fluorescence plate reader (λ_{ex} = 380 nm, λ_{em} = 445 nm).

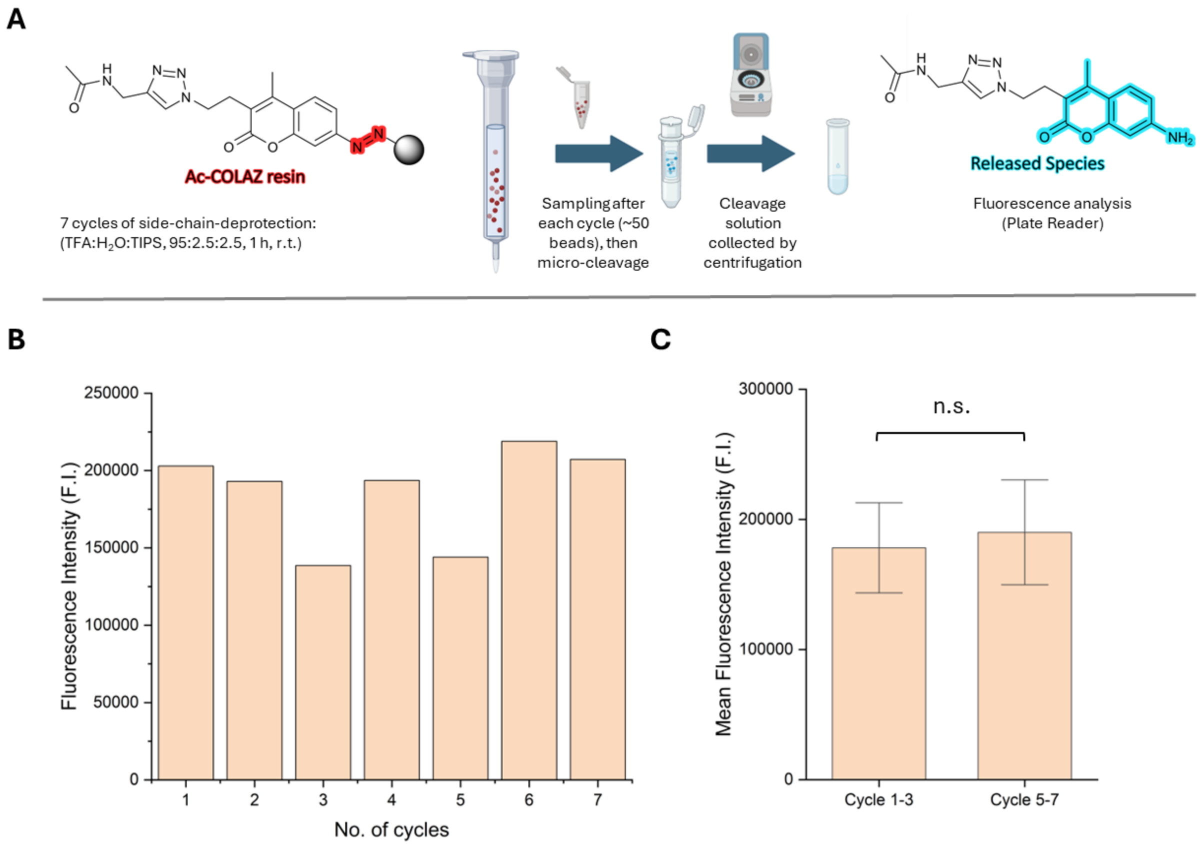

Figure S7. Acid stability studies on **Ac-COLAZ resin**. (**A**) Schematic describing the workflow. (**B**) Bar chart showing the fluorescence intensity of the released coumarin from each cycle. (**C**) Comparison of the initial and final cycles presented as mean values with error bars representing ±1 standard deviation; statistical analysis was performed using a two-sample *t*-test assuming unequal variances, with $p > 0.05$.

Synthesis of **Peptide 1** (Ac-WHISKEY-spacer-AMC)

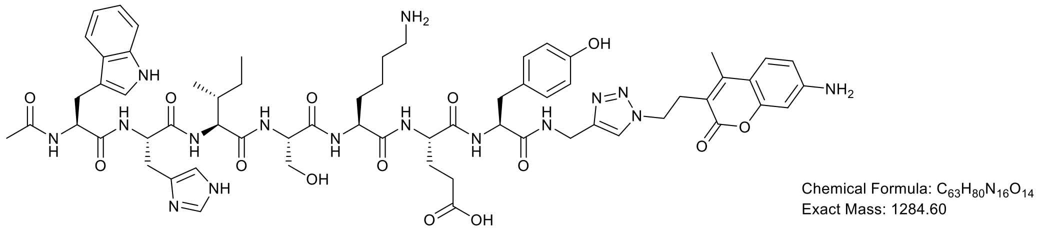

Automated SPPS was carried out in triplicate using a Liberty Blue™ peptide synthesiser. The reactor was charged with **Fmoc-COLAZ resin** (175 mg, 49.0 µmol, assumed at manufacturer's loading 0.28 mmol/g) and subjected to cycles of Fmoc-deprotection (20% morpholine in DMF), wash (3 x 3 mL, DMF) then amide coupling with Fmoc-protected amino acids Fmoc-Trp(Boc)-OH, Fmoc-His(Trt)-OH, Fmoc-Ile-OH, Fmoc-Ser(*t*Bu)-OH, Fmoc-Lys(Boc)-OH, Fmoc-Glu(O*t*Bu)-OH and Fmoc-Tyr(*t*Bu)-OH using the standard coupling protocol.

After acetylation, the side-chain protecting groups were cleaved by shaking the resin in 90:5:2.5:2.5 TFA:EDT:H_2O:TIPS (5 mL/ 175 mg resin, 2 h), then the resin was rinsed extensively with DCM, then Et_2O (4 mL, 3 x 2 min), and dried under vacuum. Deprotected peptide was cleaved from the resin using a 0.25 M sodium dithionite solution in 1:1 *t*BuOH:water (4 mL/175 mg resin, 4 x 10 min), followed by rinsing with 1:1 *t*BuOH:water (4 mL/175 mg resin, 4 x 10 min). All of the drained solutions were combined and directly lyophilised to obtain the crude product.

The crude product was purified on prep-HPLC (C18) (method A) to yield **Peptide 1** (14.2 mg, 32 % based on **Fmoc-COLAZ resin** loading of 0.21 mmol/g). HRMS (MALDI, CHCA) $[M+H]^+$ found 1285.61297, $C_{63}H_{81}N_{16}O_{14}^+$ requires 1285.61127 (1.32 ppm).

Synthesis of **Peptide 2** (Ac-WHISKEY-spacer-COLAZ)

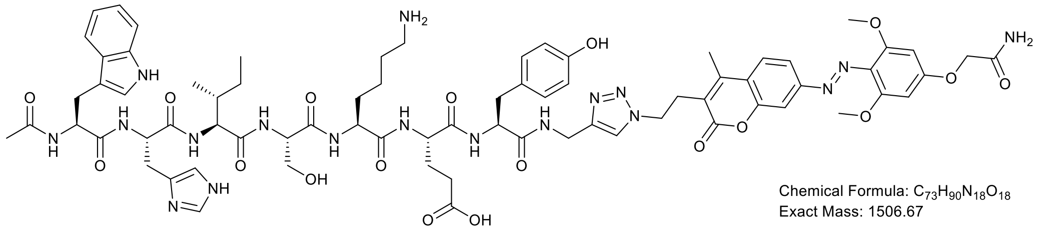

Automated SPPS was carried out in triplicate using a Liberty Blue™ peptide synthesiser. The reactor was charged with **Fmoc-COLAZ Rink AM resin** (63 mg, 50.0 µmol, assumed at manufacturer's loading 0.80 mmol/g) and subjected to cycles of Fmoc-deprotection (20% morpholine in DMF), wash (3 x 3 mL, DMF) then amide coupling with Fmoc-protected amino acids Fmoc-Trp(Boc)-OH, Fmoc-His(Trt)-OH, Fmoc-Ile-OH, Fmoc-Ser(*t*Bu)-OH, Fmoc-Lys(Boc)-OH, Fmoc-Glu(O*t*Bu)-OH and Fmoc-Tyr(*t*Bu)-OH using the standard coupling protocol.

After acetylation, global deprotection and cleavage was performed by shaking the resin in 95:2.5:2.5 TFA:H_2O:TIPS (5 mL/ 175 mg resin, 2 h) and then cleavage solution was drained and the resin washed with DCM (4 x 5 mL). The combined solutions were concentrated under a flow of nitrogen and slowly dripped into ice cold ether to precipitate the crude peptide. The crude product was purified on prep-HPLC (C18) (method A) to yield **Peptide 2** (6.13 mg, 12 % based on **Fmoc-COLAZ Rink AM resin** loading of 0.54 mmol/g). HRMS (MALDI, CHCA) $[M+H]^+$ found 1507.67432, $C_{73}H_{91}N_{18}O_{18}^+$ requires 1507.67532 (0.66 ppm).

Synthesis of **Peptide 3** (Ac-R10-spacer-AMC)

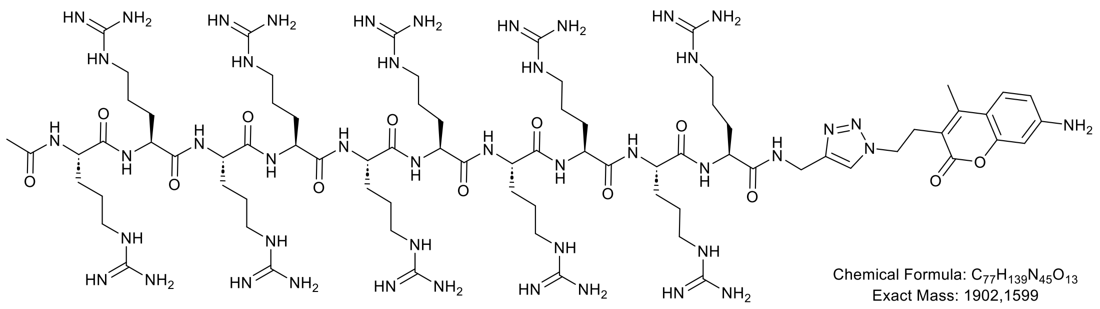

Automated SPPS was carried out in triplicate using a Liberty Blue™ peptide synthesiser. The reactor was charged with **Fmoc-COLAZ resin** (175 mg, 49.0 µmol, loading assumed at manufacturer's quote 0.28 mmol/g) and subjected to cycles of Fmoc-deprotection (20% morpholine in DMF), wash (3 x 3 mL, DMF) then amide coupling with the Fmoc-protected amino acid Fmoc-Arg(Pbf)-OH using the arginine double coupling protocol. After completion, the solution was drained, washed with DMF (4 mL, 3 x 2 min), DCM (4 mL, 3 x 2 min), then Et_2O (4 mL, 3 x 2 min), and dried under vacuum.

After acetylation, the side-chain protecting groups were cleaved by shaking the resin in 95:2.5:2.5 TFA:H_2O:TIPS (5 mL/ 175 mg resin, 4 h + 1 h + 1 h), the resin was rinsed extensively with DCM, then Et_2O (4 mL, 3 x 2 min), and dried under vacuum. Deprotected peptide was cleaved from the resin using a 0.25 M sodium dithionite solution in 1:1 *t*BuOH:water (4 mL/175 mg resin, 4 x 10 min), followed by rinsing with 1:1 *t*BuOH:water (4 mL/175 mg resin, 6 x 10 min). All of the drained solutions were combined and directly lyophilised to obtain the crude product.

The crude product was purified on prep-HPLC (C18) (method B) to yield purified **Peptide 3** (12.2 mg, 16.9 % based on **COLAZ resin** loading of 0.22 mmol/g). HRMS (MALDI, CHCA) $[M+H]^+$ found 1903.17303, $C_{77}H_{140}N_{45}O_{13}^+$ requires 1903.16718 (3.07 ppm).

UPLC Chromatograms, Peptides

Peptide 1 (Ac-WHISKEY-spacer-AMC)

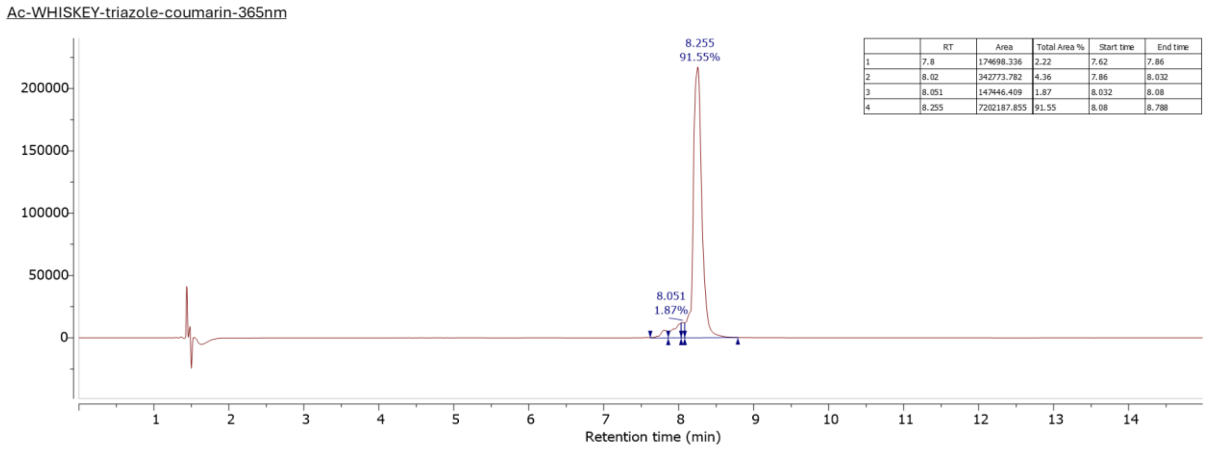

	RT	Area	Total Area %	Start time	End time
1	7.8	174698.336	2.22	7.62	7.86
2	8.02	342773.782	4.36	7.86	8.032
3	8.051	147446.409	1.87	8.032	8.08
4	8.255	7202187.855	91.55	8.08	8.788

Peptide 2 (Ac-WHISKEY-spacer-COLAZ)

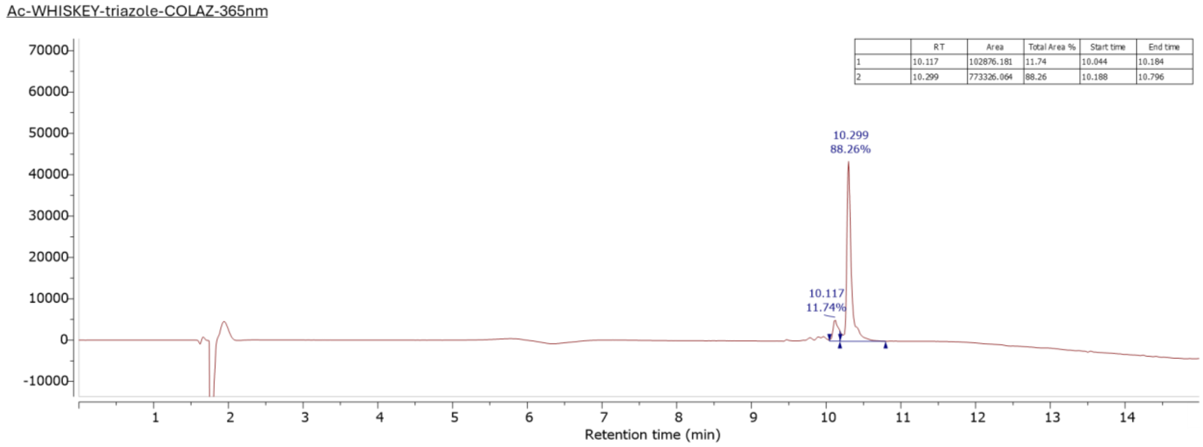

	RT	Area	Total Area %	Start time	End time
1	10.117	102876.181	11.74	10.044	10.184
2	10.299	773326.064	88.26	10.188	10.796

Peptide 3 (Ac-R10-spacer-AMC)

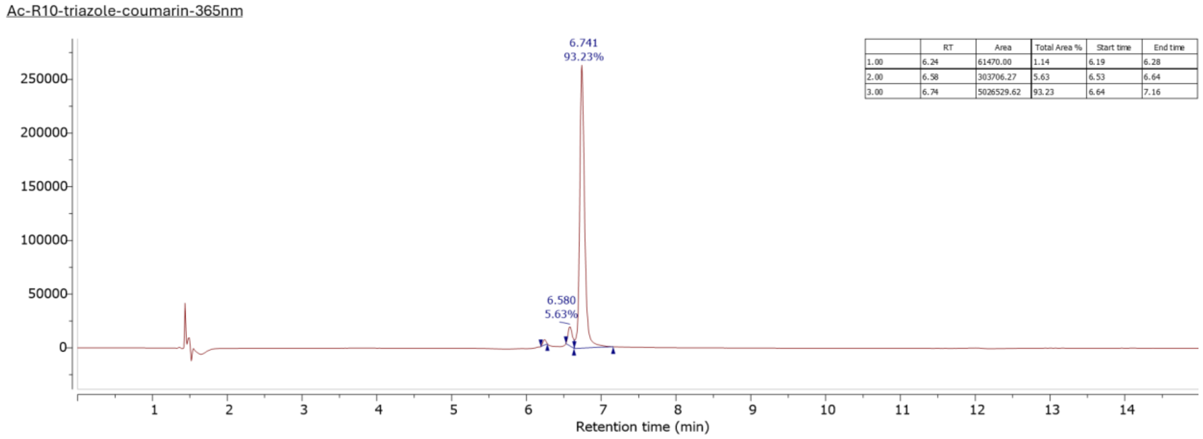

	RT	Area	Total Area %	Start time	End time
1.00	6.24	61470.00	1.14	6.19	6.28
2.00	6.58	303706.27	5.63	6.53	6.64
3.00	6.74	5026529.62	93.23	6.64	7.16

MALDI Spectra, Peptides

Peptide 1 (Ac-WHISKEY-spacer-AMC)

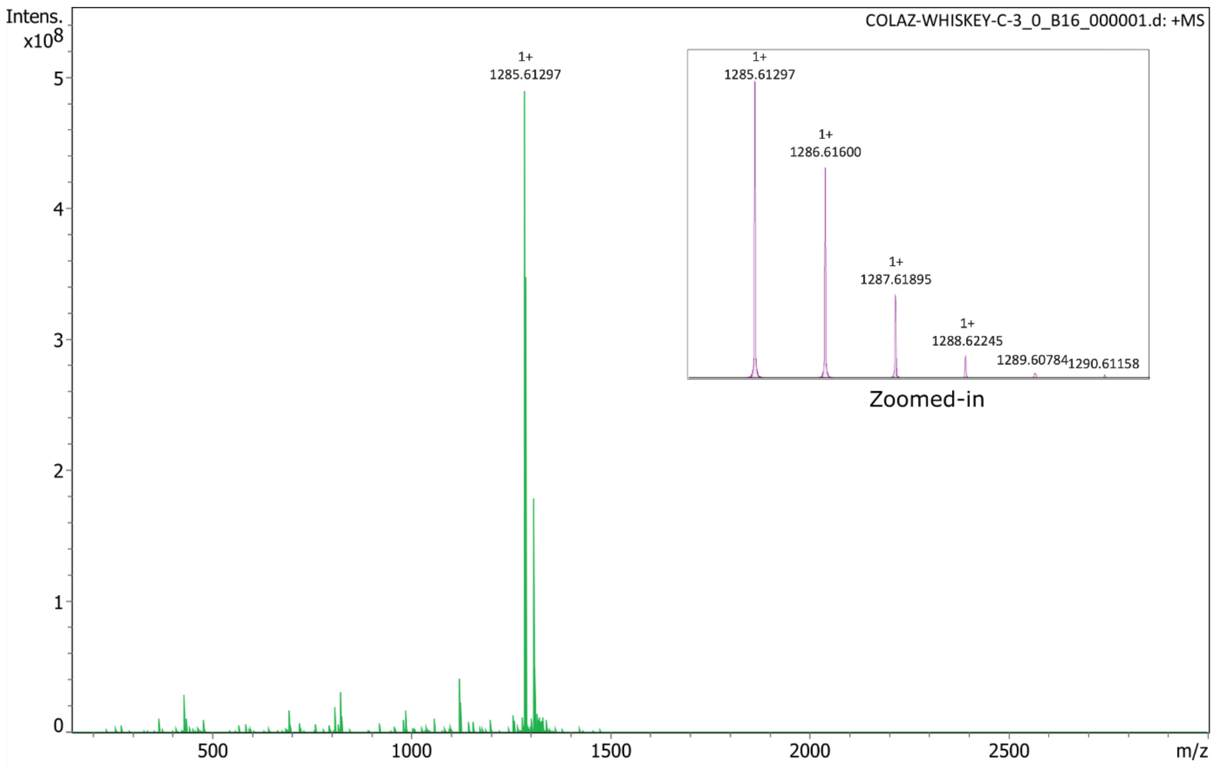

Peptide 2 (Ac-WHISKEY-spacer-COLAZ)

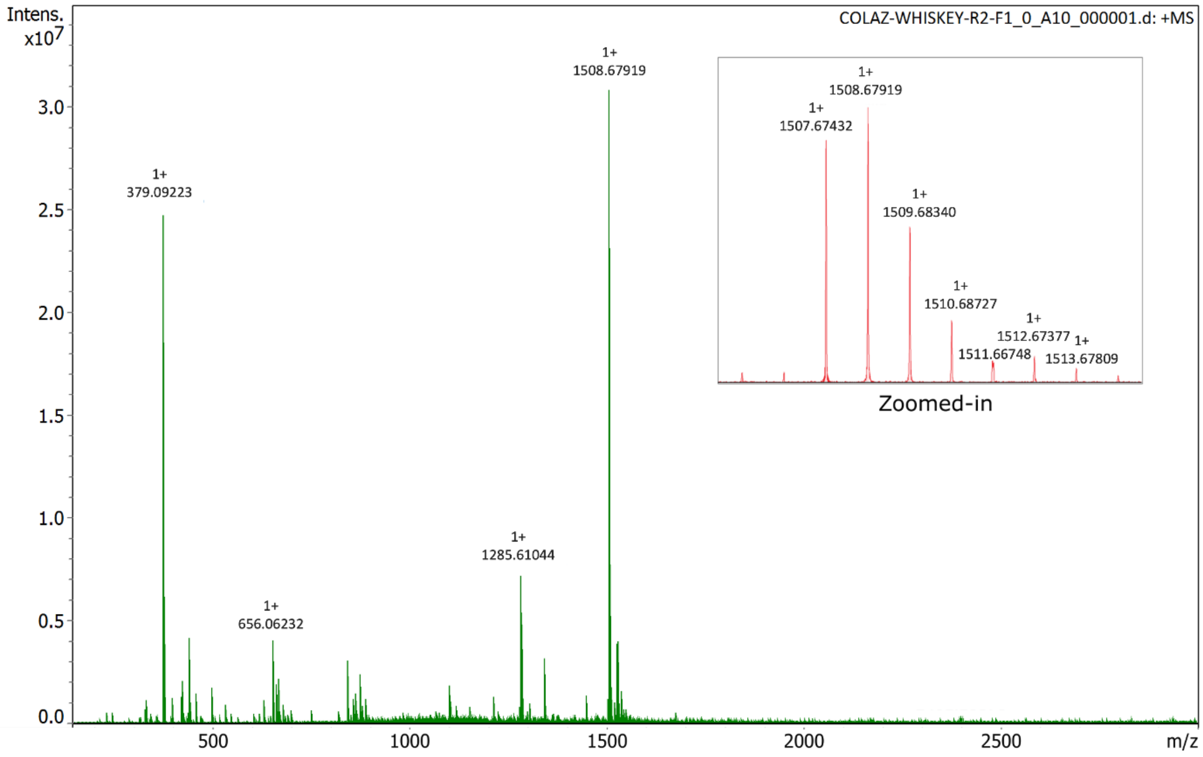

Peptide 3 (Ac-R10-spacer-AMC)

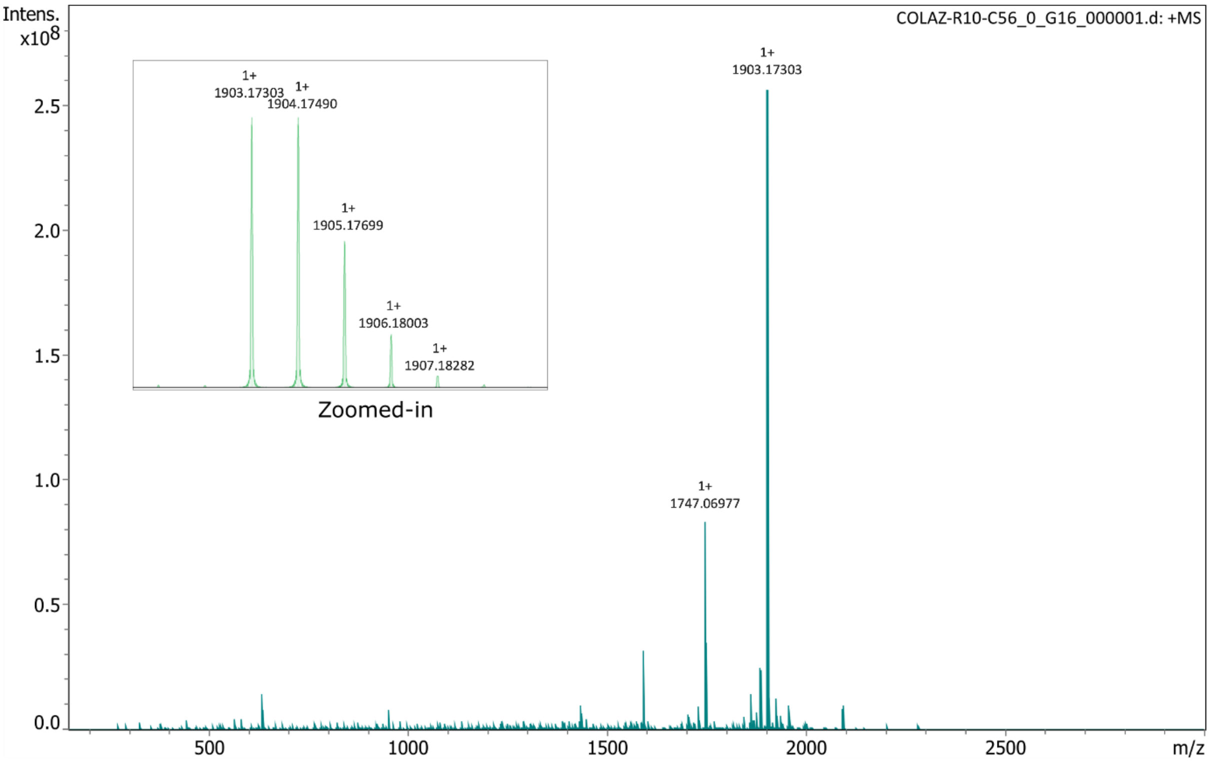

Biorthogonal Cleavage of Peptide 2

Sample preparation: A 10 mM stock solution of **Peptide 2** was prepared in DMSO. Glutathione (GSH, 50 mM) in phosphate buffered saline (PBS) was used to prepare GSH dilutions at 50, 10, 5.0, 1.0 and 0.5 mM in PBS. 198 µL of each GSH dilution was transferred to a 96-well plate and incubated for 10 mins at 37 °C. Then 2 µL of the stock solution of **Peptide 2** was quickly transferred to each well and a time course experiment was initiated in the plate reader.

Reaction Monitoring: Samples were prepared on a 96-well plate, with individual wells containing 200 µL sample. Time course monitoring of the cleavage of the COLAZ linker in **Peptide 2** was performed on a ClarioStar Plus fluorescence plate reader using top-optics laser (λ_{ex} = 365 nm, λ_{em} = 450 nm). The sampling interval was set at every 5 mins over a period of 3 hours. The plate was shaken at 300 rpm between readings and kept at 37 °C.

Table S1. Raw data for Figure 2, showing GSH-cleavage of **Peptide 2**.

	Fluorescence Intensity (F.I.)					
Time (Mins)	50 mM	10 mM	5 mM	1 mM	0.5 mM	water
0	1077	516	354	327	326	316
5	7026	1676	468	339	342	332
10	13410	3277	614	358	349	349
15	17114	5095	745	360	345	342
20	18982	6955	879	364	347	335
25	19779	8671	1025	371	346	330
30	19895	10213	1159	376	349	329
35	19915	11556	1290	375	350	324
40	19955	12746	1429	384	355	326
45	19945	13740	1576	387	357	324
50	19884	14643	1717	395	366	330
55	19930	15548	1863	399	360	324
60	19728	16054	1990	407	363	322
65	19732	16575	2123	412	360	320
70	19777	17099	2268	420	369	324
75	19677	17509	2394	426	369	321
80	19783	17834	2540	431	373	320
85	19912	18347	2683	438	373	319
90	20071	18744	2824	451	381	318
95	19990	18633	2920	450	380	317
100	19961	18880	3060	457	384	317
105	19978	18995	3183	461	386	315
110	19944	19220	3337	473	385	315
115	19994	19388	3458	476	388	313
120	20011	19410	3578	482	393	313
125	20151	19469	3708	493	395	312
130	20186	19483	3829	497	400	311
135	19791	19311	3914	499	395	307
140	19653	19295	4034	506	398	306
145	19470	19235	4142	507	396	302
150	19546	19240	4253	514	400	303
155	19536	19248	4360	519	402	298
160	19478	19275	4468	526	401	296
165	19573	19260	4591	531	406	296
170	19664	19278	4709	539	407	293
175	19555	19296	4794	540	410	290

Cell Culture and Imaging

Three mammalian cell lines were used to study the uptake of COLAZ-R10 peptides. Michigan Cancer Foundation-7 (MCF-7) cells, HeLa cervical cancer cells and murine macrophage RAW 264.7. All three cell lines were cultured under the same conditions.

<u>Cell culture</u>

Cells were cultured in Dulbecco's modified Eagle's medium (DMEM, high glucose, Thermofisher) supplemented with 10% FBS, 1% Penicilin-Streptomycin (Pen-Step), 1% L-glutamine at 37 °C in a humidified atmosphere containing 5% CO_2.

<u>Cell Passage</u>

Once the cell culture reached 70-80% confluency, the flask was aspirated, and washed with phosphate-buffered saline (PBS) (2 mL). Then the cells were trypsinised using Gibco™ Trypsin-EDTA (0.05%) for 5 mins at 37 °C in an incubator. When all the cells were fully detached, the trypsin was neutralised with supplemented DMEM (4 mL). The media was centrifuged (300 g x 3 mins) and aspirated. The cell pellet was resuspended in supplemented DMEM (1 mL), and the desired amount was transferred to a new T75 flask containing supplemented DMEM (9 mL).

<u>Cell Counting</u>

Cell solution in media was mixed with Trypan blue dye in 1:1 ratio (10 µl : 10 µl) and pipetted to a C-Slide chamber (Invitrogen™ Countess™) (10 µl in each chamber). The slide was inserted into an Invitrogen Countess 3 Automated Cell Counter, the live cell count was automatically determined from the captured brightfield image. The same procedure was repeated for the other chamber. Then the desired amount of cell solution was distributed to fluoro-dishes (FD35) aiming for 3.0×10^5 cells for next day imaging.

<u>Cryopreservation</u>

Harvested cells from a T75 flask were suspended in freezing media (supplemented-DMEM with 10 % DMSO). 1 mL of the solution was transferred to a cryo-vial (Corning) placed inside a cryogenic tub, jacket filled with isopropanol and stored at -80 °C.

<u>Frozen cell recovery</u>

Cryo-vials were rapidly warmed to room temperature and suspended in warm supplemented-DMEM (2 mL) in a falcon tube (15 mL). The cell was centrifuged (300g x 3 mins) and aspirated. The cell pellet was resuspended in supplemented-DMEM (1 mL) and transferred to a T75 flask with supplemented-DMEM (9 mL).

Cell treatment

On the day of imaging, the treatment solution was prepared in a separate 12-well plate, each well containing 3 mL of phenol-red free, supplemented-DMEM (0.5 % DMSO or R10 peptide). The plated cells in fluorodishes (FD35) were aspirated and washed with PBS, then treated with 2 mL of the compound-media solution for 1 hour at 37 °C in an incubator. After 1 hour, the cells were aspirated and washed with PBS and re-supplied with phenol-red free, supplemented-DMEM (2 mL).

Stimulated-Raman Scattering (SRS) and fluorescence Imaging

Images were captured using a custom-designed multi-modal microscope setup. A picoEmerald S laser system (APE, Berlin, Germany) supplied a tuneable pump laser (700–990 nm, 2 ps, 80 MHz repetition rate) alongside a spatially and temporally combined Stokes laser (1031 nm, 2 ps, 80 MHz repetition rate). The output beams were directed into the scanning unit of an Olympus FV1000MPE microscope through a series of dielectric mirrors and guided into an Olympus XLPL25XWMP N.A. 1.05 objective lens using a 690 nm short-pass dichroic mirror (Olympus). For SRS measurements, the Stokes beam was intensity modulated using a 20 MHz EoM integrated into the picoEmerald S. Forward scattered light was gathered with another 25× Olympus XLPL25XWMP N.A. 1.05 objective lens, with Stokes light being filtered out using a Chroma ET890/220m filter. A telescope focused this light onto an APE silicon photodiode, linked to an APE lock-in amplifier set with a 10 µs time constant. The lock-in amplifier signal was transmitted to an Olympus FV10-Analog unit. Laser powers post-objective reached 22.2 – 25.3 mW for the pump laser and 90 mW for the Stokes laser. The tuneable pump laser was set to 791.2 nm to acquire the SRS (-CH_3) images.

To acquire the fluorescence image, the tuneable pump laser was tuned to 740 nm to match the two-photon excitation of coumarin and the epidetected fluorescence emissions were separated from scattered laser light using the following filters from Semrock: FF552-Di02, FF483/639-Di01 and FF510/84 and detected on a photon multiplier tube.

Images were recorded at resolutions of 1024 × 1024 pixels with a pixel dwell time ranging from 10 µs, by averaging 2 times, utilizing Olympus's FluoView FV10-ASW scanning software. Image analysis and processing were carried out using FIJI 1.54p.[2]

[2] J. Schindelin, I. Arganda-Carreras, E. Frise, V. Kaynig, M. Longair, T. Pietzsch *et. al.*; *Nat. Methods*; 2012; **9**, 676-682.

Fluorescence Quantification

The acquired images were processed in ImageJ, The SRS (-CH_3) images were used to set a threshold for cell areas. The selected areas were copied to the fluorescence images and measurements of the mean fluorescence of the defined area were recorded. The quantified mean fluorescence units were normalised to DMSO measurements (100%).

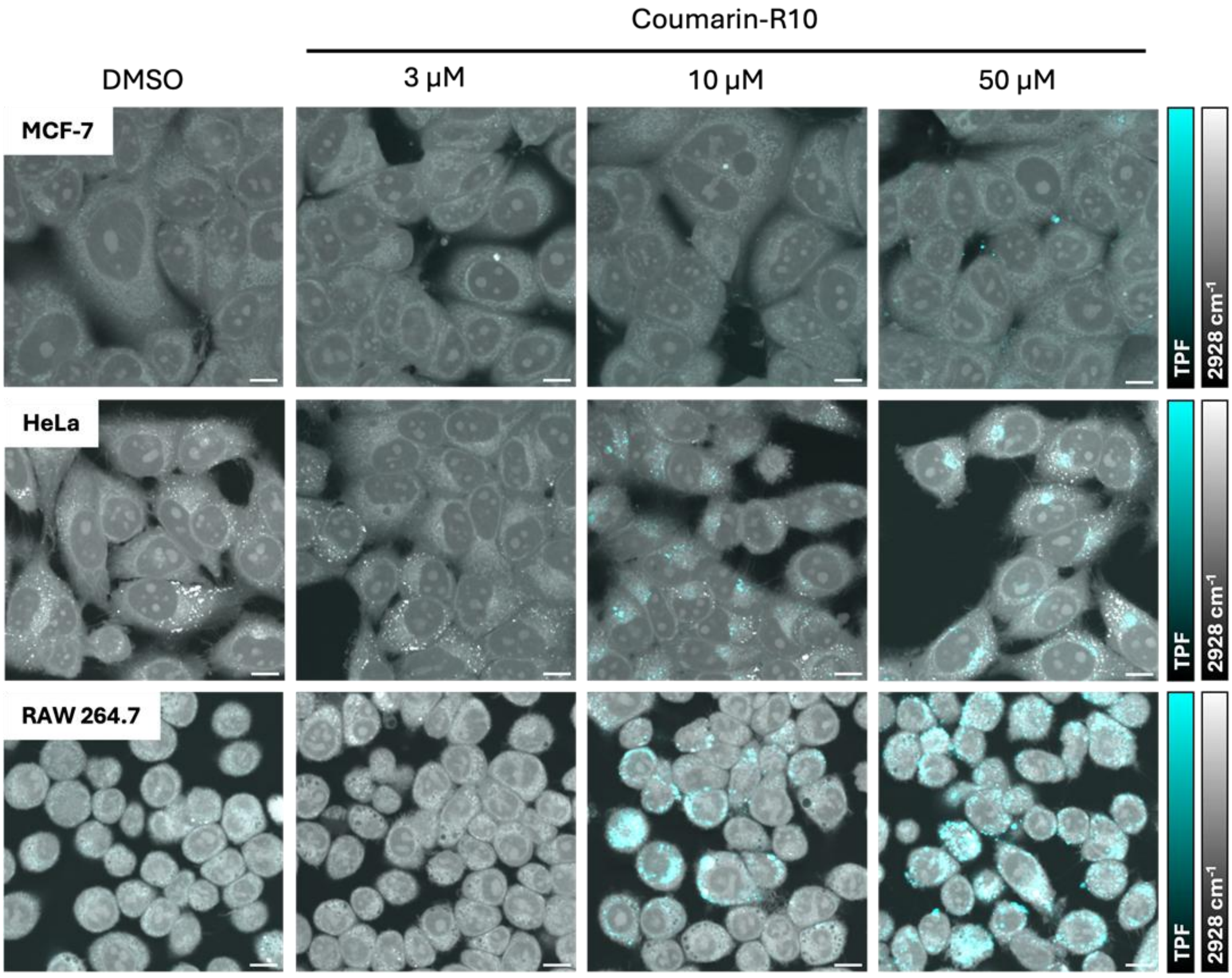

Figure S8. Stimulated Raman scattering (SRS) images (CH_3, 2928 cm^{-1}) overlaid with two-photon fluorescence images (emission collected at 520-552nm) of coumarin-labelled R10 peptides in MCF-7, HeLa and RAW264.7 cells treated with different concentrations (Scale bars = 10 μm).
